# Improved Equations for the Torsional Strength of Reinforced Concrete Beams for Codes of Practice Based on the Space Truss Analogy

**DOI:** 10.3390/ma15113827

**Published:** 2022-05-27

**Authors:** Luís F. A. Bernardo, Mafalda M. Teixeira, Dario De Domenico, Jorge M. R. Gama

**Affiliations:** 1 Centre of Materials and Building Technologies (C-MADE), Department of Civil Engineering and Architecture, University of Beira Interior, 6201-001 Covilhã, Portugal; mafalda.m.teixeira@ubi.pt; 2 Department of Engineering, University of Messina, 98166 Messina, Italy; dario.dedomenico@unime.it; 3 Center of Mathematics and Applications (CMA), Department of Mathematics, University of Beira Interior, 6201-001 Covilhã, Portugal; jgama@ubi.pt

**Keywords:** reinforced concrete, beams, torsional strength, correlation study, codes of practice, space truss model, thin-walled tube analogy

## Abstract

Design codes provide the necessary tools to check the torsional strength of reinforced concrete (RC) members. However, some researchers have pointed out that code equations still need improvement. This study presents a review and a comparative analysis of the calculation procedures to predict the torsional strength of RC beams from some reference design codes, namely the Russian, American, European, and Canadian codes for RC structures. The reliability and accuracy of the normative torsional strengths are checked against experimental results from a broad database incorporating 202 RC rectangular beams tested under pure torsion and collected from the literature. The results show that both the readability and accuracy of the codes’ equations should be improved. Based on a correlation study between the experimental torsional strengths, and geometrical and mechanical properties of the beams, refined yet simple equations are proposed to predict torsional strength. It is demonstrated that the proposed formulation is characterized by a significant improvement over the reference design codes. The efficiency of the proposed formulae is also assessed against another equation earlier proposed in the literature, and an improvement is noted as well. From the results, it can be concluded that the proposed equations in this study can contribute to a more accurate and economical design for practice.

## 1. Introduction

In engineering practice, structural members under pure torsion are not a common situation. Usually, torsional effects are combined with other internal forces in the critical sections of the member. However, there are several practical cases in structures in which structural members must sustain primary torsional effects in their critical sections. Typical examples are encountered in bridge structures or geometrically complex building structures, in which reinforced concrete (RC) girders and columns can be subjected to primary torsion due to the high eccentricity of static loads or complex geometry of the members (e.g., curvature in plan). For such members, an accurate calculation of the torsional strength is essential for the design or assessment of the torsional capacity, namely to guarantee or check the safety at the ultimate limit states. For such purposes, current RC members and structural engineers usually base their calculations on the rules from design codes. Nowadays, each country has its own set of design codes that govern structural design, namely for RC structures, and some of them have a high impact in other countries and also constitute reference codes for the international community [1,2,3,4,5,6,7].

Despite all the research effort made in recent decades by the scientific community, several current design codes for concrete structures are still somewhat scarce in providing detailed and specific design rules for torsion. These include basic reinforcement detailing rules and limits for important design variables to ensure a good performance of RC members under torsion for both the ultimate and serviceability limit states. For example, some design codes do not provide any specific rule regarding the minimum amount of torsional reinforcement, which is considered a basic requirement to avoid a sudden failure after concrete cracking. The same can be stated for the maximum amount of torsional reinforcement to ensure ductility at failure (torsional reinforcement should yield before concrete crushing). Although such a maximum amount can be indirectly computed from the maximum compressive stress allowed for the concrete struts, this upper-stress limit can vary substantially among design codes. As there is a lack of specific rules for torsion, some codes refer to the rules related to the reinforcement requirements for other internal forces, such as for bending (for the longitudinal reinforcement) and for shear (for the transverse reinforcement). In addition to the aforementioned missing aspects, when the rules from several design codes are used to predict the torsional strength of RC beams, small accuracy and high dispersion of the results, including unsafe predictions, are still observed when they are compared with experimental data [8,9]. This observation shows that research work on the torsion of RC beams still needs to be carried out to propose more accurate design rules to be incorporated in future revisions of design codes.

The first reference design codes, which incorporated specific design rules for torsion for RC members, were based on the so-called skew-bending theory. This model was proposed by Hsu in 1968 [10] and was established from empirical observations based on the failure pattern observed in several experiments with RC rectangular beams under pure torsion. This model showed to provide accurate predictions for the torsional strength of RC beams with common rectangular cross-sections, such as the ones used in building structures. However, when applied to cross-sections with large aspect ratios or to cross-sections with more complex geometries, such as the ones used in bridge girders, this model produces more complex formulations and shows to be much less accurate. In spite of this, the skew-bending theory was developed over more than two decades [11,12,13] and had a considerable influence on the design rules for torsion in some reference design codes, such as the ACI code (American code) up to 1995. Presently, the design rules for torsion of the Eurasian code SiNiP 2018 [1] are still based on the skew-bending theory.

Nowadays, the torsion design rules from most design codes for concrete structures are based on the space truss analogy. This model, first proposed by Raush in 1929 [14], was later combined with the classical thin tube theory from Bredt [15] and further developed in the second half of the last century [16,17,18,19]. The space truss analogy allows for a better physical understanding of how an RC beam behaves under torsion in the cracked stage and provides simple equations to compute the torsional strength, even for geometrically complex cross-sections. However, due to different hypotheses incorporated in the model, to allow for a simple torsional design, the calculation procedures for torsion can be somewhat different among the design codes. Because of this, noticeable differences can be observed in the results when different design codes are used to predict the torsional strength of RC beams, although all these codes are formally based on the same space truss resisting mechanism. These observations justify the need for additional improvements to be incorporated in future revisions of the codes.

Since the 1980s, refined versions of models based on the space truss analogy have been proposed that allow one to compute with accuracy the strength of RC beams under pure torsion [18,20,21,22,23,24,25,26], RC beams under torsion combined with other internal forces [27,28,29,30]. More advanced analytical models have also been proposed in the literature and applied to beams under torsion and combined loadings [31,32,33,34,35,36]. Although these models have been shown to be very reliable when compared with experimental results, they are not easy to be used by practitioners as they require advanced calculation procedures to be implemented on the computer. Hence, simple and reliable equations would be preferable for practice.

Based on these motivations and research needs, this study first presents a critical overview and a comparative analysis of the calculation procedures from design codes to predict the torsional strength of RC rectangular beams. For this, some reference design codes considered important due to their territorial scope were chosen. Such design codes are the following ones: the Eurasian code, SiNiP 2018 [1], two different versions of American codes, namely ACI 318R-89 [2] and ACI 318R-19 [3], the European codes MC90 [4], MC10 [5] and Eurocode 2 [6], and the Canadian code CSA A23.3-14 [7]. For comparison purposes, this list includes codes based on different mechanical models to establish the design rules for torsion, namely the skew-bending theory and the space truss analogy, and also codes with different application scopes (laws or recommendation documents). The calculation procedures from the codes are summarized and checked against a broad database incorporating 202 RC rectangular beams tested under pure torsion collected from the literature. This database includes under- and over-reinforced beams, plain and hollow beams, as well as normal- and high-strength concrete beams. Then, based on correlations studies, improved and simple equations are proposed to compute the torsional strength of RC beams. The proposed model correlates the torsional strength and three main properties of the beams: the compressive concrete strength, the concrete area enclosed within the outer perimeter of the cross-section, and the amount of torsional reinforcement. The accuracy and reliability of the proposed equations are checked against the results from the reference design codes. They are also checked against simple equations proposed by Rahal in 2013 [8], which have a similar form to the ones proposed here and were based on a similar approach to that used in this study (by fitting experimental results). For these reasons, the research from Rahal [8] was considered a benchmark. The results show that the proposed equations significantly improve the accuracy and reliability of the torsional strength of RC beams when compared with the same ones from the reference design codes. They also give slightly better results when compared with the ones from the equations previously proposed by Rahal [8].

When compared to previous research, namely the one from Rahal [8], which constitutes a reference study, the main novelty in this study is related to the higher number of reference design codes studied, the higher number of reference beams considered in the database, and the somewhat different methodology used to fit the experiment results in obtaining simple and improved equations for torsional strength.

It should be noted that this article addresses only the particular case of RC beams under pure torsion. It is well known that in real concrete structures, the critical section of members usually carries combined loadings, for instance, torsion combined with other internal forces (bending, shear, and axial forces). However, for some concrete members, such as girders curved in plan and girders with eccentric loadings, torsion could be the primary internal acting force. Furthermore, the design provisions to check the interaction between internal forces requires the calculation of the torsional strength of the cross-section, considering only torsion as loading. Hence, the ultimate strength of the cross-section needs to be well known. This justifies the importance of this study.

## 2. Equations from the Reference Design Codes

From the studied design codes, the SiNiP 2018 [1] and ACI 318R-89 [2] codes are the only ones whose equations for torsional design, i.e., to compute the torsional strength of RC beams, are based on the skew-bending theory. Although ACI 318R-89 is no longer in use, it was included in this study for comparison with SiNiP 2018 (which is still in use) and to better understand the influence of the underlying mechanical model in the code formulation. The calculation procedure for torsion for all the other reference design codes (ACI 318R-19 [3], MC90 [4], MC10 [5], Eurocode 2 [6], and CSA A23.3-14 [7]) are based on the space truss analogy. As far as the European design codes are concerned, it should be stated that MC10 substituted MC90. However, as the design rules for torsion were simplified in MC10, MC90 was also considered for comparison.

Table A1 in Appendix A summarizes the equations incorporated in each reference design code for torsional design and to compute the torsional strength of RC beams. In Table A1, all codes’ equations were rewritten to uniformize the symbology for better clarity and to facilitate comparison. The meaning of the used symbology can be found in the Nomenclature.

American and Canadian codes incorporate specific rules to design both the longitudinal and transverse torsional reinforcement. In general, the European codes incorporate specific equations to design longitudinal torsional reinforcement. However, they refer to the rules for shear reinforcement to design the transverse torsional reinforcement.

From the presented equations in Table A1, one can highlight five main parameters that can be defined by somewhat different rules but strongly influence the magnitude of the calculated torsional strength:

- The limit of the wall’s thickness of the equivalent hollow beam, which determines and limits the area enclosed within the flow of shear stress acting on the beam’s cross-section;
- The flow of shear stress, which is induced by the external torque, and the corresponding shear resultant forces in each wall;
- The criteria to compute the torsional strength, which, depending on the underlying mechanical model and design code, consider separately the strength contributed by the torsional tensile reinforcement and the strength contributed by the compressive concrete;
- The maximum limit allowed for the compressive stress in the concrete struts to avoid a brittle failure of the beam due to concrete crushing;
- The angle of the concrete struts to the longitudinal axis of the beam.

A more detailed analysis of the summarized equations in Table A1 can be found in some of the reference design codes and also in a previous study from the two first authors [9].

## 3. Database with Reference Beams

For this study, an extensive literature review was performed to compile the main properties and experimental results of RC rectangular beams tested under pure torsion until failure. A total of 202 beams were compiled from several studies [10,12,19,37,38,39,40,41,42,43,44,45,46,47,48,49] to build the database (with 158 plain beams and 44 hollow beams). The number of beams found in the literature was higher than 202; however, some of them were disregarded based on the following criteria:

1. The main properties of the tested RC beams needed to compute the normative torsional strength should be given;
2. The experimental torsional strength should be given and the RC beams should have failed in pure torsion in their ultimate stage as expected;
3. The beams should comply with reinforcement requirements from ACI 318R-19 [3]. For instance, and among other requirements, the spacing of the hoops should be less than the upper limit set by the code to avoid untypical behavior (for instance, premature failure) during testing. ACI 318R-19 [3] was the chosen code because it was found to be the one that incorporates a higher number of specific detailing rules for RC beams under torsion.

Table A2 in Appendix A summarizes the main geometric and mechanical properties of the reference RC beams that are necessary to compute the torsional strength from the design codes. The meaning of each parameter can be found in the Nomenclature.

**Figure 1:**
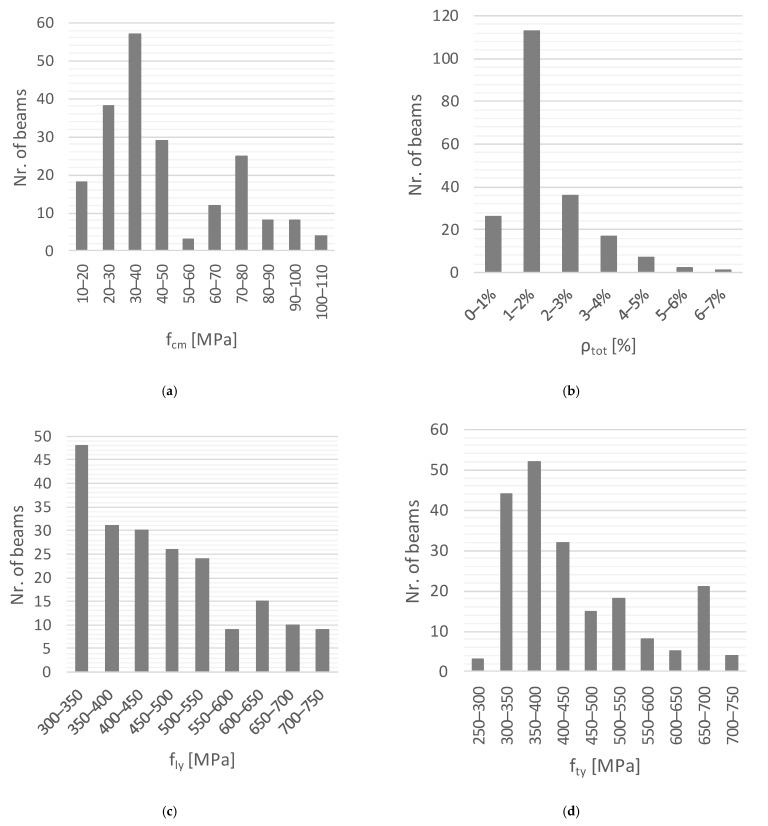
Distribution of key parameters for the reference RC beams. (**a**) Concrete strength. (**b**) Total reinforcement ratio. (**c**) Yielding stress of longitudinal reinforcement. (**d**) Yielding stress of transverse reinforcement.

Figure 1 presents graphs with the distribution of some key parameters for the 202 reference RC beams from the database. In the abscissa of the graphs, the parameters are: $f_{cm}\;$is the average compressive concrete strength, $\rho_{tot}=\rho_{l}+\rho_{t}\;$is the total ratio of torsional reinforcement, $f_{ly}$ and $f_{ty}$ are the average yielding stresses for longitudinal and transverse reinforcement, respectively.

Figure 1 shows that 142 and 60 beams are built with normal- (up to 50 MPa) and high-strength concrete (over 50 MPa, according to [5]), respectively. The average concrete compressive strength ranges between 14 MPa and 110 MPa. The total reinforcement ratio ranges between a minimum of 0.37% and a maximum of 6.36%, being for most of the beams in the range of 1 to 2%. The yielding stress ranges between 308.8 MPa and 723.9 MPa for the longitudinal reinforcement and between 285 MPa and 714.8 MPa for the transverse reinforcement. For most of the beams, it ranges between 300 MPa and 500 MPa.

The database used in this study is wider than the ones used in previous studies on the torsion of RC beams. For instance, the database used by Rahal [8], which is an important reference for this study, included 50 beams less than the database used in this study.

## 4. Evaluation of Design Codes

For each reference beam from the database (see Table A2), the theoretical torsional strength, $T_{R,th}$, was computed according to the calculation procedures from each reference design code (see Table A1). The obtained values are presented in Table A3 in Appendix A, which also presents the corresponding experimental values, $T_{R,exp}$. The calculated ratios $T_{R,exp}/T_{R,th}$ are presented in a Table A4 in Appendix A. Table 1 summarizes, for each design code, the average value, $\bar{x}$, and the coefficient of variation, *cv*, computed for the ratios, $T_{R,exp}/T_{R,th}$, from all reference beams. The results are presented separately for plain beams (P), hollow beams (H), and also for all beams together (P + H). This separation can be justified because some design codes include corrections to the equations for hollow beams, while others do not.

From Table 1, it can be stated that all design codes show a relatively high dispersion for the ratio $T_{R,exp}/T_{R,th}$ (in general *cv* > 20%), which represents a motivation for developing more accurate and reliable torsional strength equations.

Design codes based on the skew-bending theory, namely Si-NiP 2018 [1] and ACI 318R-89 [2] codes, present similar results. In general, they both tend to underestimate the torsional strength, ($\bar{x}$ > 1), with $\bar{x}$ = 1.25 for Si-NiP 2018 code and $\bar{x}$ = 1.15 for ACI 318R-89 code. Among these two design codes, the ACI 318R-89 code shows the best results, with $\bar{x}$ closer to 1 and less dispersion of the results disregarding the cross-section type (with *cv* = 24%, against *cv* = 32% for SiNiP 2018 code). Regarding the cross-section type, it can be observed that the accuracy of the ACI 318R-89 code seems to be better for plain beams (with $\bar{x}$ = 1.12 and *cv* = 23% for plain beams and $\bar{x}$ = 1.27 and *cv* = 26% for hollow beams); similar trends are observed for Si-NiP 2018 code (with $\bar{x}$ = 1.21 and *cv* = 34% for plain beams and $\bar{x}$ = 1.47 and *cv* = 20% for hollow beams). This observation can be explained because the model based on the skew-bending theory was calibrated for plain beams [10].

The other design codes based on the space truss analogy (ACI 318R-19 [3], MC90 [4], MC10 [5], Eurocode 2 [6], and CSA A23.3-14 [7]) show results with some differences among them. Among those design codes, and disregarding the cross-section type, the CSA A23.3-14 code seems to be the most accurate (with $\bar{x}$ = 1.01 and *cv* = 24%), while the MC10 code seems to be one of the worst (with $\bar{x}$ = 1.33 and *cv* = 44%). ACI 318R-19, MC90, and Eurocode 2 codes show the same level of dispersion (with *cv* = 28% for all of them), and all tend to underestimate the torsional strength (with $\bar{x}$ ranging from 1.33 and 1.40). Regarding the cross-section type, it can be observed that the CSA A23.3-14 code is the only one that tends to slightly overestimate the torsional strength of hollow beams (with $\bar{x}$ = 0.98 and *cv* = 22 %). Eurocode 2 seems to be one that provides the best results for plain sections (with $\bar{x}$ = 1.07 and *cv* = 24%). For hollow beams, it is not so clear because of the higher dispersion of the results.

It is also worth noting that the ACI 318R-89 code (currently not in use) provides, in general, more accurate results and with less dispersion when compared with the ACI 318R-19 code. This is because the majority of the reference tested beams have small rectangular cross-sections, for which the skew-bending theory provides better results (as previously referred).

Based on the above considerations and balancing the accuracy with the degree of safety, it can be concluded that, among the codes currently in use, Eurocode 2 seems to be the one that presents the most satisfactory results. However, some caution is required with this conclusion (and other ones previously stated) as the dispersion of the results is high for all codes.

**Figure 2:**
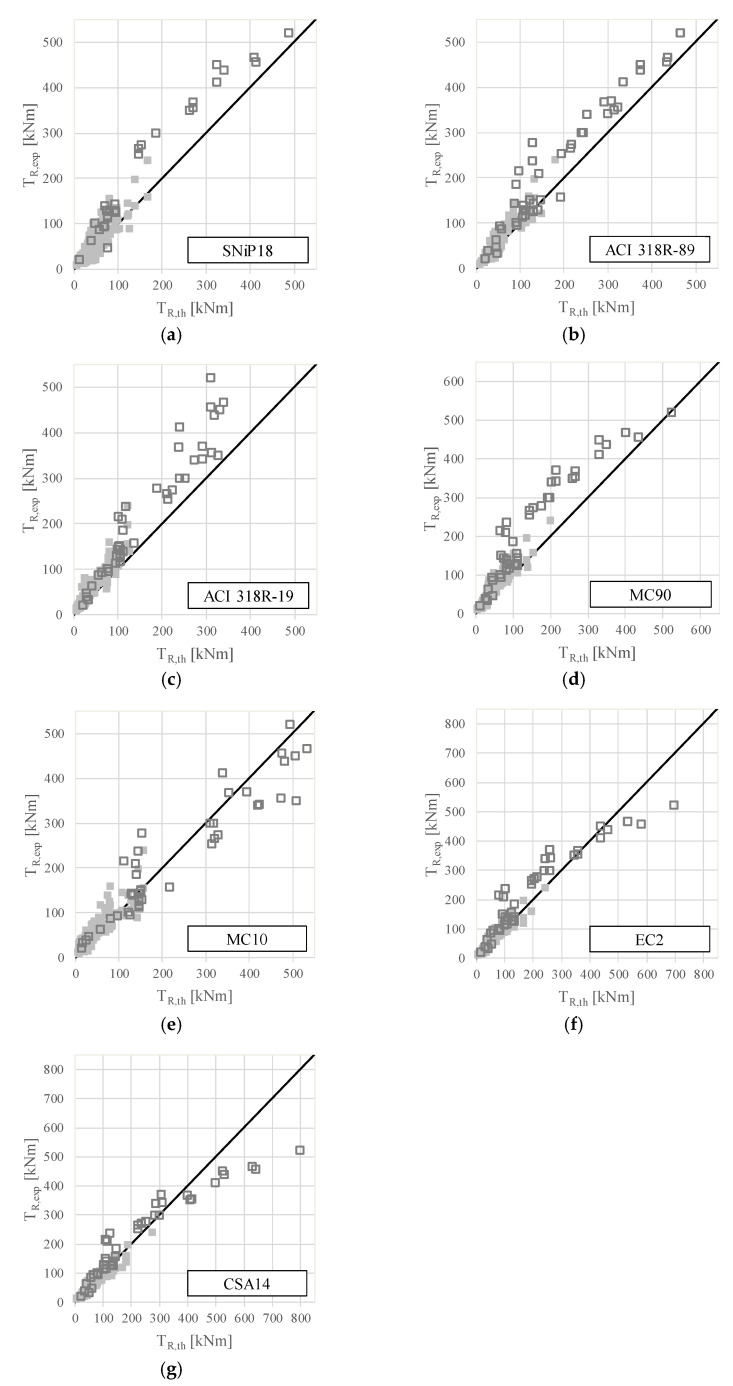
Experimental vs. theoretical torsional strengths (design codes). (**a**) SNiP18. (**b**) ACI 318R_89. (**c**) ACI 318R_19. (**d**) MC90. (**e**) MC10. (**f**) EC2. (**g**) CSA14.

The predictive accuracy of each code formulation can be assessed in Figure 2, which presents scatter plots relating to the experimental torsional strengths (in ordinate) with the theoretical ones (in abscissa) for each of the reference codes. In the graphs, different markers were used to distinguish the results for plain beams (“⬛”) and for hollow beams (“⬜”).

In each graph, an inclined line with a 45° angle is drawn, which represents the location of the points in case both the experimental and theoretical torsional strengths are equal, i.e., the code predicts exactly the torsional strength of the beam. Points located on the left side of the referred line correspond to the case in which the code underestimates the torsional strength of the beams. If the points are located on the right side of the line, then the code overestimates the torsional strength.

From Figure 2, it can be seen that some of the design codes can overestimate the torsional strength of several reference beams noticeably, in particular, for hollow beams. This is the case for the MC10 and CSA A23.3-14 codes.

The results from Table 1 and Figure 2 show clearly that the level of accuracy of all analyzed codes, as well as the level of safety for some of them, should be improved.

## 5. Equations Proposed by Rahal 

Rahal, in 2013 [8], showed that for both the ACI and CSA codes, after some basic algebraic manipulations, a general and simple torsional strength equation can be written in the form of Equation (1). This equation is written here with some parameters standardized according to the nomenclature used in the code equations previously presented. Equation (1) was used in some previous models [18,23].

(1) $$T_{R}=2A_{k}\sqrt{\frac{A_{l}f_{yl}}{u_{k}}\frac{A_{t}f_{yt}}{s}}\;$$

It can be shown that the general form of Equation (1) can be obtained from all the code equations, which are based on the space truss analogy. This is the case of the reference codes considered in this study (ACI 318R-19 [3], MC90 [4], MC10 [5], Eurocode 2 [6], and CSA A23.3-14 [7])).

Rahal [8] pointed out the following drawbacks for Equation (1) based on experimental evidence:

1. In the hollow tube model used in the space truss analogy, the shear flow is constant around the perimeter of the tube walls. Design code formulations consider a constant effective thickness for all the walls. As a consequence, the model assumes the same shear stress and shear. As pointed out by Rahal [8], this is not consistent with the experimental results on RC rectangular beams under torsion that show different conditions on the different faces of the cross-section [10,12,37]. Experiments show that larger tensile strains are observed in the longer legs of the hoops and larger diagonal strains are observed in the longer faces of the cross-section. In this regard and based on these observations, a refined variable-angle space truss model incorporating different strut inclination angles in the different faces of the cross-section was recently proposed by De Domenico [50];
2. Rahal [8] also pointed out that Equation (1) disregards the effect of the concrete compressive strength, while experiments [10,12] show that this parameter has a significant influence on the torsional strength;
3. In addition, Rahal [8] also noted that in most experiments [17,40,51], the concrete of the beams did not spall at the maximum torque or was limited to the corners of the cross-section [37,42]. However, in Equation (1), the torsional strength is related to the spalled concrete dimensions through area $A_{k}$ (area enclosed within the shear flow path).

To solve the first drawback, Rahal [8] proposed to reduce the power by 0.5 for the reinforcement term in Equation (1) to compensate for the relatively smaller contribution of the hoops and concrete on the shorter side of the cross-section. To solve the second drawback, the author suggested incorporating an additional term to consider the effect of the concrete compressive strength in Equation (1). For the third drawback, the author simply suggested correcting Equation (1) to relate the torsional strength with the unspalled concrete dimensions, substituting the reduced area, $A_{k}$, with the concrete area, $A_{c}$ (area enclosed within the outer perimeter of the cross-section), and the corresponding perimeter is denoted as $p_{c}$ (in place of $p_{h}$).

Based on the experimental results collected from the literature (which included 152 RC beams tested under torsion) and based on separated nonlinear correlations (using appropriate subsets of the reference beams) for each of the previously referred terms/parameters to correct Equation (1), Rahal [8] proposed the improved Equations (2) and (3) to compute the torsional strength of RC beams. These equations are written here according to the nomenclature and metric units for the parameters used in this study (as referred to in Section 6):

(2) $$T_{R}=0.33{\left(f_{c}\right)}^{0.16}A_{c}{\left(A_{l}f_{yl}\frac{A_{t}f_{yt}}{s}\right)}^{0.35}$$

(3) $$\leq 2500{\left(f_{c}\right)}^{0.3}\frac{A_{c}{}^{2}}{p_{c}}$$

It should be noted that, in Equation (2), the power for the concrete strength term was empirically selected by Rahal [8] to provide good results.

Equation (2) governs the torsional strength for under-reinforced sections (the failure is governed by the yielding of the torsional reinforcement) and includes the “reinforcement term” ($A_{l}f_{yl}A_{t}f_{yt}/s)$ and the “concrete strength term” (a term related to $f_{c}$). The upper limit stated in Equation (3) governs the torsional strength for over-reinforced sections (the failure is governed by concrete crushing before reinforcement yielding) and includes the “concrete strength term.” Equations (2) and (3) are not limited to rectangular cross-sections and can be applied to arbitrary cross-section shapes.

Rahal [8] checked the results from Equations (2) and (3) against the experimental results from the 152 test specimens and very good agreement was observed. In addition, a comparison with the ACI and CSA codes showed that the proposed equations provide better results, with higher accuracy and much less dispersion. The author also showed that such good results were observed for both normal- and high-strength concrete beams, as well as for under- and over-reinforced beams.

**Figure 3:**
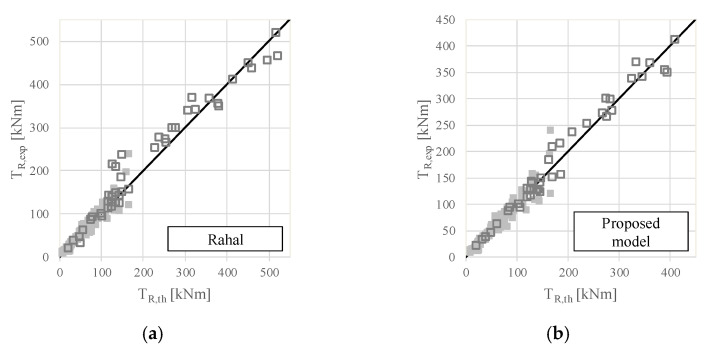
Experimental vs. theoretical torsional strengths (improved equations). (**a**) Model from Rahal. (**b**) Proposed model.

For this study, the torsional strengths computed from Equations (2) and (3) are rechecked against the experimental results of all 202 RC beams included in the wider database built for this research. The obtained results are presented for each reference beam in Table A3 and the respective ratios $T_{R,exp}/T_{theo}^{Rahal}$ are presented in Table A4. The results are summarized in Table 2 and Figure 3, in the same way as previously presented in Table 1 and Figure 2. The obtained results still confirm the conclusions from Rahal [8], namely that Equations (2) and (3) provide accurate results (with $\bar{x}$ = 1.06) with a very acceptable dispersion (with *cv* = 15%). Table 2 also shows that the results for both plain and hollow sections are very similar.

## 6. Alternative Improvement of the Equations from Rahal

In this section, Equation (1) to compute the torsional strength of RC beams is improved based on the wider database built for this study and also on a somewhat different correlation methodology than the one used by Rahal [8], namely for under-reinforced beams. The performed studies are presented separately for under- and over-reinforced beams.

### 6.1. Upper Limit to Control Concrete Crushing (Over-Reinforced Beams)

**Figure 4:**
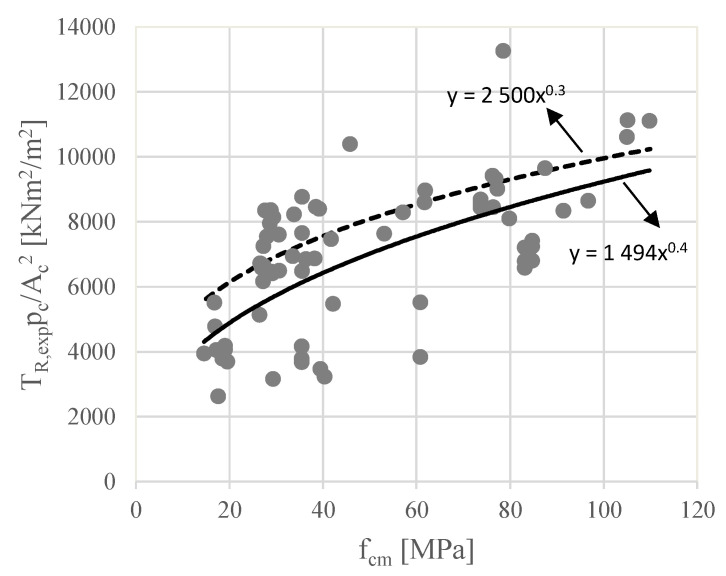
Torsional strength in over-reinforced beams.

Following the same methodology from Rahal [8] to refine the upper limit stated in Equation (3) to control concrete crushing, a subset of 70 beams (62 plain beams and 8 hollow beams) was created from the database. The failure of such beams was governed by concrete crushing in the struts without yielding the torsional reinforcement. These beams are marked with an asterisk in Table A3 and represent beams with fragile failure. For these beams, a scatter plot is presented in Figure 4, with the average concrete compressive strength ($f_{cm}$) in abscissa and the factor $T_{R,exp}p_{c}/A_{c}{}^{2}$ in ordinate (with the following units: $T_{R,exp}$ [kNm], $p_{c}$ [m], and $A_{c}$ [m^2^]). From the scatterplot, a power trendline was computed to fit the data ($1494f_{c}^{0.4}$). In the same graph, the power trendline computed by Rahal [8] and based on less reference beams is also plotted for comparison ($2500f_{c}^{0.3}$, see upper limit stated in Equation (3)). This power trendline is slightly shifted up when compared to the power trendline computed from the scatter plot in Figure 4. After computing, the torsional strengths for the over-reinforced beams from the database using an equation based on $1494f_{c}^{0.4}$ and after a comparative analysis with the experimental strengths, it was observed that more unsafe values were obtained for the reference beams (the predicted torsional strength is higher than the real one for more beams, i.e., more points are located above the trend curve). This observation can be explained due to the high dispersion observed for the points in the scatter plot in Figure 4. For practical design, this situation is not acceptable and a correction of the power trendline was studied. The results suggested that the power trendline should be slightly shifted up to minimize the referred unsafe predictions. After some attempts, it was concluded that the power trendline suggested by Rahal [8] was quite appropriate. For this reason, the power trendline $2500f_{c}^{0.3}$ was also adopted in this study and the upper limit stated in Equation (3) remained unchanged to define the upper limit to control concrete crushing (see upper limit stated in Equation (7)).

### 6.2. Reinforcement, Concrete Strength, and Concrete Area Terms (Under-Reinforced Beams)

For under-reinforced beams, Rahal [8] studied separated nonlinear correlations using appropriate subsets of reference beams from its database to study the influence of both the “reinforcement term” ($A_{l}f_{yl}A_{t}f_{yt}/s)$ and the “concrete strength term” (related to $f_{c}$). Additional explanations of the approach followed by the referred author can be found in [8]. In this study, a different correlation methodology was used. Considering 132 reference under-reinforced beams from the database, a correlation was studied between the torsional strength and three terms. Two terms are the ones referred to previously, namely the “reinforcement term” and the “concrete strength term”. In addition, a third term was added and related to the concrete area enclosed within the external perimeter of the cross-section, the so-called “concrete area term” (related to $A_{c}$). From preliminary analysis, it was found that by adding this term the correlation is noticeably improved.

As Equation (1) is linearizable with a logarithmic transformation, a multiple linear regression was performed. Applying a logarithmic transformation and adding the additional “concrete area term,” Equation (1) can be rewritten in the following general linear form:

(4) $$ln(T_{R})=A+B\;ln\left(f_{c}\right)+C\;ln\left(A_{c}\right)+D\;ln\left(A_{l}f_{yl}\frac{A_{t}f_{yt}}{s}\right),$$

where *A*, *B*, *C,* and *D* are numerical coefficients to be determined.

To perform the multiple linear regression, the data and terms for each reference beam from the database ($T_{R}$,$\;f_{c}$, $A_{c}$, and $A_{l}f_{yl}\frac{A_{t}f_{yt}}{s}$) were previously log-transformed. Then, a multiple linear model was fitted, obtaining a high R^2^ equal to 0.984, and a mean squared error equal to 0.013552. For this analysis, IBM SPSS software version 28 was used. The obtained linear fitted model is the following:

(5) $$ln(T_{R})=0.087+0.218\;ln\left(f_{c}\right)+1.013\;ln\left(A_{c}\right)+0.318\;ln\left(A_{l}f_{yl}\frac{A_{t}f_{yt}}{s}\right)$$

From the previous equation, the equivalent Equation (6) can be written to compute the torsional strength for under-reinforced beams.

### 6.3. Proposed Equations

From the results obtained in the previous subsections, Equations (6) and (7) are proposed as an improvement for Equation (1) to compute the torsional strength of RC beams. Equation (6) constitutes an alternative to Equation (2) proposed by Rahal in 2013 [8]. In Equations (6) and (7), the units of parameters are: $f_{c}$ [MPa], $A_{c}$ [m^2^], $A_{l}$ [cm^2^], $f_{yl}$ [MPa], $A_{t}$/s [cm^2^/m], $f_{yl}$ [MPa], $A_{c}$ [m^2^], and $p_{c}$ [m]. The torsional strength $T_{R}$ is computed in units [kNm].

As for Equations (2) and (3), Equation (6) governs the torsional strength for under-reinforced beams, while the upper limit stated in Equation (7) governs the torsional strength for over-reinforced beams, respectively. Equations (6) and (7) can also be applied to arbitrary cross-section shapes.

(6) $$T_{R}=1.091{\left(f_{c}\right)}^{0.218}{\left(A_{c}\right)}^{1.013}{\left(A_{l}f_{yl}\frac{A_{t}f_{yt}}{s}\right)}^{0.318}$$

(7) $$\leq 2500{\left(f_{c}\right)}^{0.3}\frac{A_{c}{}^{2}}{p_{c}}\;,$$

The results from Equations (6) and (7) are checked against the results from all 202 tested beams from the database. The torsional strengths computed from the previous equations for each reference beam ($T_{T,th}^{Prop}$) are presented in Table A3, and the ratios $T_{R,exp}/T_{T,th}^{Prop}$ are presented in Table A4. The results are summarized in Table 2 and Figure 3. They show that Equations (6) and (7) provide accurate results (with $\bar{x}$ = 1.01) with a very acceptable dispersion (with *cv* = 13%). The results are also good for both plain and hollow RC beams. A comparison with the results from the reference codes used in this study (Table 1 and Figure 2) shows that the proposed equations provide much better results, with higher accuracy and much less dispersion. When compared with the results from Equations (2) and (3) from Rahal [8], it can be concluded that they are quite similar, although the results from the equations proposed in this study are slightly better. It should also be noted that these good results were observed for both normal- and high-strength concrete beams, as well as for under- and over-reinforced beams.

Finally, Table 2 also summarizes the obtained results substituting Equation (6) with a simplified version, Equation (8). Table 2 shows that, despite the very small changes in the powers and the numerical factors, the results show that the computed torsional strengths tend to be slightly unsafe (with $\bar{x}$ = 0.96 < 1.00). This shows that the model is highly sensitive to the precision of the numerical values (numerical factor and powers).

(8) $$T_{R}=1.09{\left(f_{c}\right)}^{0.22}A_{c}{\left(A_{l}f_{yl}\frac{A_{t}f_{yt}}{s}\right)}^{0.32}$$

(9) $$\leq 2500{\left(f_{c}\right)}^{0.3}\frac{A_{c}{}^{2}}{p_{c}}\;,$$

Although it is not discussed in this paper, it is worth noting that the calibration of appropriate safety factors for material parameters as well as of a model uncertainty factor $\gamma_{Rd}$ for Equations (6) and (7) would produce a code-formatted design capacity equation compliant with a predefined reliability level [52], which could be used in the design of RC beams failing in torsion.

## 7. Conclusions

In this study, a review and comparative analysis of the calculation procedures to compute the torsional strength of RC beams from some reference design codes was performed. For this, a wide database was built, incorporating the experimental torsional strengths of 202 RC rectangular beams tested until failure and found in the literature. In addition, based on the reference RC beams from the database and on correlation studies between the torsional strength and some properties (amount of reinforcement, concrete strength, and concrete area enclosed within the external perimeter of the cross-section), simple equations to compute the torsional strength were proposed and checked.

From the obtained results, the following main conclusions can be drawn:

- In general, equations from the studied reference codes still need improvements to increase the accuracy and reduce the observed statistical dispersion;
- Some reference design codes overestimate the torsional strength of several reference RC beams from the database noticeably, which is not acceptable for design and justifies further improvements;
- The proposed equations to compute the torsional strength of RC beams showed to be much more reliable and accurate in comparison with code’s equations;
- The proposed equations are simple and can easily be used for practice to assess with accuracy the torsional strength of RC members, including plain and hollow beams, normal- and high-strength concrete beams, as well as under- and over-reinforced beams;
- When compared with similar equations from a previous study [8], the proposed equations were shown to be slightly better at predicting torsional strength;
- This study confirms that simple and reliable design equations can be obtained by simply fitting the results with experimental data existing in the literature and related to the torsional strength of the RC beams.

## Figures and Tables

**Table 1:** Comparative analysis between design codes.

Cross Section		P	H	P + H
		$T_{R,exp}/T_{R,th}$	$T_{R,exp}/T_{R,th}$	$T_{R,exp}/T_{R,th}$
SiNiP 2018 [1]	$\bar{x}$ =	1.21	1.47	1.25
	*cv* =	34%	20%	32%
ACI 318R-89 [2]	$\bar{x}$ =	1.12	1.27	1.15
	*cv* =	23%	26%	24%
ACI 318R-19 [3]	$\bar{x}$ =	1.40	1.38	1.40
	*cv* =	31%	18%	28%
MC90 [4]	$\bar{x}$ =	1.28	1.61	1.36
	*cv* =	24%	29%	28%
MC10 [5]	$\bar{x}$ =	1.41	1.07	1.33
	*cv* =	44%	33%	44%
Eurocode 2 [6]	$\bar{x}$ =	1.07	1.29	1.12
	*cv* =	24%	31%	28%
CSA A23.3-14 [7]	$\bar{x}$ =	0.98	1.13	1.01
	*cv* =	22%	27%	24%

**Table 2:** Comparative analysis for the torsional strength from improved equations.

Cross-Section		P	H	P + H
		$T_{R,exp}/T_{R,th}$	$T_{R,exp}/T_{R,th}$	$T_{R,exp}/T_{R,th}$
Rahal [8]	$\bar{x}$ =	1.05	1.08	1.06
	*cv* =	14%	17%	15%
Equations (6) and (7)	$\bar{x}$ =	1.01	1.01	1.01
	*cv* =	14%	9%	13%
Equations (8) and (9)	$\bar{x}$ =	0.96	0.96	0.96
	*cv* =	15%	9%	14%

## Data Availability

Not applicable.

## Nomenclature

$A_{c}$	area enclosed within the outer perimeter of the cross-section	$u_{k}$	perimeter of the area $A_{k}$
$A_{k}$	area within the centerline of wall’s thickness (assumed to coincide with the shear flow path)	V	alied shear force
$A_{l}$	total area of longitudinal reinforcement	$V_{R}$	resistance shear force
$A_{l1}$	area of bottom longitudinal reinforcement	$V_{Rc}$	shear force due to the compressive stresses resisted by concrete
$A_{l2}$	area of tensile longitudinal steel near the vertical face	$V_{Rl}$	shear force due to the axial stresses resisted by the longitudinal reinforcement
$A_{o}$	area enclosed within circular shear flow	$V_{R,max}$	maximum resistance shear force
$A_{oh}$	area enclosed within centerline of hoops	$V_{Rt}$	shear force due to shear stresses resisted by the transverse reinforcement
$A_{t}$	area for one bar of the hoop	x	smaller dimension of the cross-section
$f_{c}$	average compressive strength of concrete	$x_{1}$	smaller dimension of hoops
$f_{ck}$	characteristic value of the compressive strength of concrete at 28 days	y	larger dimension of the cross-section
$f_{yl}$	average yield strength of longitudinal reinforcement	$y_{1}$	larger dimension of hoops
$f_{yt}$	average yield strength of tranerse reinforcement	$z_{i}$	length of wall *i*, equal to the distance between the centerline intersection of adjacent walls
$k_{c}$	concrete reduction factor	α	angle of the hoops to the longitudinal axis
$p_{c}$	gross perimeter of the cross-section	$\alpha_{cw}$	coefficient to account for the stress state of the compressed chord
$p_{h}$	perimeter of the centerline of hoop	$\alpha_{t}$	efficiency coefficient
q	shear stress flow due to torsion	$\gamma_{c}$	partial safety factor for concrete properties
s	longitudinal spacing between hoops	*δ*	numerical coefficient to account for imperfections
T	applied torsional moment	θ	angle of the diagonal compressive stresses in concrete struts to the longitudinal axis
t	Wall’s thickness of the equivalent hollow section	*ν*	strength reduction factor for cracked concrete
$T_{c}$	torsional moment resisted by concrete	$\rho_{l}$	ratio of longitudinal reinforcement:$\;\rho_{l}=A_{l}/xy$
$T_{max}$	maximum torsional mome	$\rho_{t}$	ratio of transverse reinforcement: $\rho_{t}=A_{t}u/As$
$T_{R}$	torsional moment resistance	τ	shear stress due to torsion
$T_{R,exp}$	experimental torsional moment resistance	ϕ	reduction coefficient
$T_{R,th}$	theoretical torsional moment resistance	$\phi_{c}$	concrete resistance factor
$T_{s}$	torsional moment resisted by the reinforcement	$\phi_{s}$	reinforcement resistance factor
$T_{u}$	ultimate torsional moment		

## Appendix A

**Table A1:** Equations from the reference design codes.

	SinNiP 2018	ACI 318R-89
Limit of the section wall thickness	Not available	Wall thickness of the hollow section: If t≥x/4 the section is considered as a plain section; If x/10t≤t<x/4 the section is considered as an equivalent plain section.
Shear stresses and shear forces due to torsion	Not available	Not available
Safety condition	Condition: $T_{R}\leq T_{max}$ where $T_{R}=min\left\{T_{R1};T_{R2}\right\}$ $T_{R1}=0.5A_{l1}f_{yl}y+A_{t}f_{yt}\left(\frac{x^{2}y}{s\left(2y+x\right)}\right)$ $T_{R2}=0.5A_{l2}f_{yl}x+A_{t}f_{yt}\left(\frac{y^{2}x}{s\left(2x+y\right)}\right)$	Condition: $T_{u}\leq T_{R}$ where $T_{R}=T_{c}+T_{s}$ For plain section: $T_{c}=0.8{\left(f_{c}\right)}^{1/2}x^{2}y$ For hollow section: $T_{c}=0.8{\left(f_{c}\right)}^{1/2}x^{2}y\left(\frac{4t}{x}\right)$ $T_{s}=\frac{A_{t}}{s}\alpha_{t}x_{1}y_{1}f_{ty}$ where $\alpha_{t}=0.66+0.33\left(y_{1}/x_{1}\right)\leq 1.5$ $\mathrm{and}\;x_{1}\leq y_{1}$
Maximum limit by the tension in concrete struts	Maximum limit: $T_{max}=0.1f_{c}x^{2}$y	Maximum limit: $\mathrm{If}\;T_{s}\leq 4T_{c}$, then $T_{R}=T_{c}+T_{s}$ $\mathrm{If}\;T_{s}>4T_{c}$, then $T_{R}=5T_{c}$
*	Not available	Not available

* Angle between the concrete struts and the longitudinal axis of the beams.

	**ACI 318R-19**	**MC 90**
Limit of the section wall thickness	Wall thickness of the hollow section: If $t\geq A_{oh}/p_{h}$ the section is considered as a plain section; If $t<A_{oh}/p_{h}$ the section is considered as an equivalent plain section.	Wall thickness of the hollow section: If $t=A/u\leq t_{real}$ the section is considered as a hollow section; If $t=A/u>t_{real}$ the section is considered as a plain section (equivalent hollow section).Wall thickness of the plain section (equivalent hollow section): t=A/u and t> 2x distance between the face of the section and the axis of the longitudinal reinforcement.
Shear stresses and shear forces due to torsion	Shear flow for a thin-walled tube: q=τt Shear stress along the perimeter of the section: $\tau =\frac{T}{2A_{o}t}$ with $A_{o}=0.85A_{oh}$	Shear flow at a wall i: $\tau_{i}t_{i}=\frac{T}{2A_{o}\delta }$ Shear stress at a wall i: $V_{i}=\frac{Tz_{i}}{2A_{o}\delta }$ where $\delta =1.0-0.25\left(x/y\right)$, with y≥x.
Safety condition	Condition: $T_{u}\leq \phi T_{R}$ where ϕ=1 (for the present study). $T_{R}=min\left\{\left(a\right);\left(b\right)\right\}$ $\left(a\right)\;T_{R}=2A_{o}\frac{A_{t}}{s}f_{yt}\cos\theta$ $\left(b\right)\;T_{R}=2A_{l}f_{yl}\frac{\tan\theta }{p_{h}}$	Condition: $V_{R}=min\left\{\left(a\right);\left(b\right);\left(c\right)\right\}$ (a) Shear force due to the force on the longitudinal reinforcement: $V_{Rl}\leq A_{l}f_{yl}/\cot\theta$ (c) Shear force due to the force on the transverse reinforcement: $V_{Rt}=A_{t}f_{yt}u_{k}\cot\theta /s$
Maximum limit by the tension in concrete struts	Maximum limit to plain section: $T_{u}\leq \phi 8{\left(f_{c}\right)}^{1/2}1.7\frac{A_{oh}^{2}}{p_{h}}$ Maximum limit to hollow section: $T_{u}\leq \phi 8{\left(f_{c}\right)}^{1/2}1.7A_{oh}t$ where ϕ=1 (for the present study).	Maximum limit: Shear force due to the force on the concrete struts: $V_{Rc}\leq f_{cd2}tu_{k}\cos\theta \sin\theta$ where $f_{cd2}=0.60\left(1-f_{ck}/250\right)f_{c}$
*	$\cot^{2}\theta =\frac{A_{l}f_{yl}s}{A_{t}f_{yt}p_{h}}$	$\cot^{2}\theta =\frac{A_{l}f_{yl}s}{A_{t}f_{yt}u_{k}}$
* Angle between the concrete struts and the longitudinal axis of the beams.		

	**MC 10**	**Eurocode 2**
Limit of the section wall thickness	Wall thickness of the hollow section: $t=t_{real}$ Wall thickness of the plain section (equivalent hollow section): t= 2x distance between the face of the section and the axis of the longitudinal reinforcement and t≤x/8.	Wall thickness of the hollow section: If $t=A/u\leq t_{real}$ the section is considered as a hollow section; If $t=A/u>t_{real}$ the section is considered as a plain section (equivalent hollow section).Wall thickness of the plain section (equivalent hollow section): t=A/u e t> 2x distance between the face of the section and the axis of the longitudinal reinforcement.
Shear stresses and shear forces due to torsion	Shear stress at a wall i: $V_{i}=\frac{Tz_{i}}{2A_{k}}$	Shear flow at a wall i: $\tau_{i}t_{i}=\frac{T}{2A_{k}}$ Shear stress at a wall i: $V_{i}=\tau_{i}t_{i}z_{i}$
Safety condition	Condition: $V_{R}=V_{Rc}+V_{Rt}\leq V_{R,max}$ *Approximation level II:* $V_{Rc}=0$ $V_{Rt}=\frac{A_{t}}{s}u_{k}f_{yt}\cot\theta$	Condition: $\frac{T}{T_{R,max}}\leq 1.0$ $V_{R}=\frac{A_{t}}{s}u_{k}f_{yt}\cot\theta$
Maximum limit by the tension in concrete struts	Maximum limit: $V_{R,max}=k_{c}\left(\frac{f_{ck}}{\gamma_{c}}\right)tu_{k}\left(\frac{\cot\theta -\cot\alpha }{1+\cot^{2}\theta }\right)$ Approximation level II: $k_{c}=0.55{\left(30/f_{ck}\right)}^{1/3}\leq 0.55$ and α=90º	Maximum limit: $T_{R,max}=2\nu \alpha_{cw}f_{c}A_{k}t\sin\theta \cos\theta$ where $\nu =0.6\left[1-\left(f_{ck}/250\right)\right]$ and $\alpha_{cw}=1$, for non-prestressed beams.
*	$\cot^{2}\theta =\frac{2A_{l}f_{yl}s}{A_{t}f_{yt}u_{k}}$	$\cot^{2}\theta =\frac{A_{l}f_{yl}s}{A_{t}f_{yt}u_{k}}$
* Angle between the concrete struts and the longitudinal axis of the beams.		

	**CSA A23.3-14**
Limit of the section wall thickness	Wall thickness of the hollow section: If $t\geq A_{oh}/p_{h}$ the section is considered as a plain section; If $t<A_{oh}/p_{h}$ the section is considered as an equivalent plains section.
Shear stresses and shear forces due to torsion	Not available
Safety condition	Condition: $T_{u}\leq T_{R}$ where $T_{R}=2A_{o}\phi_{s}\frac{A_{t}}{s}f_{yt}\cos\theta$ with $A_{o}=0,85A_{oh}$ and $\phi_{s}=1$ (for the present study).
Maximum limit by the tension in concrete struts	Maximum limit to plain section: $T_{u}\leq 0.25\phi_{c}f_{c}1.7\frac{A_{oh}^{2}}{p_{h}}$ Maximum limit to hollow section: $T_{u}\leq 0.25\phi_{c}f_{c}1.7A_{oh}t$ where $\phi_{c}=1$ (for the present study).
*	$\cot^{2}\theta =\frac{A_{l}f_{yl}s}{0.45A_{t}f_{yt}p_{h}}$
* Angle between the concrete struts and the longitudinal axis of the beams.	

**Table A2:** Geometric and mechanical properties of the reference RC beams.

Ref.	Beam	**	x	y	t	$x_{1}$	$y_{1}$	$A_{l1}$	$A_{l2}$	$A_{l}$	$A_{t}/s$	$f_{c}$	$f_{yl}$	$f_{yt}$
			(m)	(m)	(m)	(m)	(m)	(cm^2^)	(cm^2^)	(cm^2^)	(cm^2^/m)	(MPa)	(MPa)	(MPa)
Hsu (1968)	B1	P	0.254	0.381	-	0.216	0.343	2.53	2.53	5.07	4.68	27.6	314.0	341.0
	B3	P	0.254	0.381	-	0.216	0.343	5.73	5.73	11.36	10.16	28.1	327.6	320.0
	B4	P	0.254	0.381	-	0.216	0.343	7.74	7.74	15.48	14.01	29.2	320.0	323.4
	B5	P	0.254	0.381	-	0.216	0.343	10.20	10.20	20.39	18.47	30.6	332.4	321.4
	B6	P	0.254	0.381	-	0.216	0.343	12.90	12.90	25.81	22.58	28.8	331.7	322.8
	B7	P	0.254	0.381	-	0.216	0.343	2.53	2.53	5.16	10.16	26.0	320.0	318.6
	B8	P	0.254	0.381	-	0.216	0.343	2.53	2.53	5.16	22.58	26.8	322.1	320.0
	B9	P	0.254	0.381	-	0.216	0.343	5.73	5.73	11.36	4.66	28.8	319.3	342.8
	B10	P	0.254	0.381	-	0.216	0.343	12.90	12.90	25.8	4.66	26.5	334.0	342.0
	C2	P	0.254	0.254	-	0.216	0.216	2.53	2.53	5.07	6.07	26.5	334.0	345.0
	C4	P	0.254	0.254	-	0.216	0.216	5.73	5.73	11.36	13.11	27.2	336.6	327.6
	C5	P	0.254	0.254	-	0.216	0.216	7.74	7.74	15.48	17.67	27.2	328.3	329.0
	C6	P	0.254	0.254	-	0.216	0.216	10.20	10.20	20.39	23.91	27.6	315.9	327.6
	G2	P	0.254	0.508	-	0.216	0.470	3.97	3.97	7.94	5.91	30.9	323.0	334.0
	G3	P	0.254	0.508	-	0.216	0.470	5.73	5.73	11.36	8.29	26.8	338.6	327.6
	G4	P	0.254	0.508	-	0.216	0.470	7.74	7.74	15.48	11.29	28.3	325.5	321.4
	G5	P	0.254	0.508	-	0.216	0.470	10.20	10.20	20.39	15.05	26.9	331.0	327.6
	G6	P	0.254	0.508	-	0.216	0.470	2.53	3.80	7.60	5.61	29.9	334.0	350.0
	G7	P	0.254	0.508	-	0.216	0.470	3.97	5.96	12.00	8.84	31.0	319.3	322.8
	G8	P	0.254	0.508	-	0.216	0.470	5.73	8.60	17.03	12.32	28.3	322.1	329.0
	I2	P	0.254	0.381	-	0.216	0.343	3.97	3.97	7.94	7.25	45.2	325	349
	I3	P	0.254	0.381	-	0.216	0.343	5.73	5.73	11.36	10.16	44.8	343.4	333.8
	I4	P	0.254	0.381	-	0.216	0.343	7.74	7.74	15.48	14.01	45.0	315.2	326.2
	I5	P	0.254	0.381	-	0.216	0.343	10.20	10.20	20.39	18.47	45.0	310.3	325.5
	I6	P	0.254	0.381	-	0.216	0.343	12.90	12.90	25.81	22.58	45.8	325.5	329.0
	J1	P	0.254	0.381	-	0.216	0.343	2.53	2.53	5.16	4.66	14.3	327.6	346.2
	J2	P	0.254	0.381	-	0.216	0.343	3.97	3.97	8.00	7.21	14.6	320.0	340.7
	J3	P	0.254	0.381	-	0.216	0.343	5.73	5.73	11.36	10.16	16.9	388.6	337.2
	J4	P	0.254	0.381	-	0.216	0.343	7.74	7.74	15.48	14.01	16.8	324.1	331.7
	K2	P	0.152	0.495	-	0.114	0.457	2.53	3.80	7.74	6.77	30.6	335.9	337.9
	K3	P	0.152	0.495	-	0.114	0.457	3.97	5.96	12.00	10.42	29.0	315.9	320.7
	K4	P	0.152	0.495	-	0.114	0.457	5.73	8.60	17.03	15.05	28.6	344.1	340.0
	M1	P	0.254	0.381	-	0.216	0.343	3.97	3.97	8.00	4.76	29.9	326.2	353.1
	M2	P	0.254	0.381	-	0.216	0.343	5.73	5.73	11.36	6.77	30.6	329.0	357.2
	M3	P	0.254	0.381	-	0.216	0.343	7.74	7.74	15.48	9.24	26.8	322.1	326.2
	M4	P	0.254	0.381	-	0.216	0.343	10.20	10.20	20.39	12.33	26.6	318.6	326.9
	M5	P	0.254	0.381	-	0.216	0.343	12.90	12.90	25.81	15.63	28.0	335.2	331.0
	M6	P	0.254	0.381	-	0.216	0.343	10.20	15.30	30.58	15.63	29.4	317.9	340.7
	N1	P	0.152	0.305	-	0.130	0.283	1.43	1.43	2.84	3.50	29.5	352.4	341.4
	N1a	P	0.152	0.305	-	0.130	0.283	1.43	1.43	2.84	3.50	28.7	346.2	344.8

** P—Plain section; H—Hollow section.

**Ref.**	**Beam**	******	x	y	t	$x_{1}$	$y_{1}$	$A_{l1}$	$A_{l2}$	$A_{l}$	$A_{t}/s$	$f_{c}$	$f_{yl}$	$f_{yt}$
			**(m)**	**(m)**	**(m)**	**(m)**	**(m)**	**(cm^2^)**	**(cm^2^)**	**(cm^2^)**	**(cm^2^/m)**	**(MPa)**	**(MPa)**	**(MPa)**
	N2	P	0.152	0.305	-	0.130	0.283	2.53	2.53	5.16	6.35	30.4	331.0	337.9
	N2a	P	0.152	0.305	-	0.130	0.283	2.53	2.53	1.61	6.21	28.4	333.1	360.7
	N3	P	0.152	0.305	-	0.130	0.283	1.43	2.14	4.26	5.08	27.3	351.7	351.7
	N4	P	0.152	0.305	-	0.130	0.283	2.53	3.25	6.58	7.98	27.3	340.9	355.9
[17]	T4	P	0.500	0.500	-	0.454	0.454	5.65	5.65	18.10	10.28	35.3	356.7	356.7
Leonhardt, Schelling (1974)	VB2	P	0.440	0.240	-	0.420	0.220	2.63	1.46	7.01	5.84	26.4	541.4	541.4
	VB3	P	0.440	0.240	-	0.420	0.220	2.63	1.46	7.01	5.84	39.1	541.4	541.4
	VB4	P	0.440	0.240	-	0.420	0.220	2.63	1.46	7.01	5.84	49.8	541.4	541.4
	VM1	P	0.294	0.160	-	0.280	0.146	1.50	1.00	3.00	3.63	39.1	442.4	568.9
	VM2	P	0.440	0.240	-	0.420	0.220	3.30	2.20	6.60	5.32	36.1	431.6	436.5
	VM3	P	0.587	0.320	-	0.561	0.294	6.42	4.28	12.84	7.14	40.0	461.0	442.4
	VQ1	P	0.324	0.324	-	0.304	0.304	1.15	1.15	3.46	2.88	19.0	557.1	557.1
	VQ3	P	0.580	0.186	-	0.560	0.166	1.83	0.61	4.27	3.05	17.6	432.6	432.6
	VQ9	P	0.806	0.140	-	0.786	0.120	2.54	0.56	5.08	2.82	19.5	441.4	441.4
	VS2-VQ2	P	0.440	0.240	-	0.420	0.220	1.53	0.92	3.66	3.05	19.0	432.6	432.6
	VS3	P	0.440	0.240	-	0.420	0.220	2.14	1.22	5.49	4.55	19.5	432.6	432.6
	VS4-VQ5	P	0.440	0.240	-	0.420	0.220	2.75	1.53	7.32	6.10	19.0	432.6	432.6
	VS9	P	0.440	0.240	-	0.420	0.220	1.16	1.16	3.48	2.90	17.6	570.9	570.9
	VS10-VB1	P	0.440	0.240	-	0.420	0.220	2.61	1.45	6.96	5.80	19.0	570.9	570.9
	VU1	P	0.440	0.240	-	0.420	0.220	1.40	0.84	3.36	5.60	19.5	441.4	441.4
	VU2	P	0.440	0.240	-	0.420	0.220	1.96	1.12	5.04	5.60	19.5	441.4	441.4
	VU3	P	0.440	0.240	-	0.420	0.220	2.52	1.40	6.72	4.18	18.5	441.4	441.4
	VU4	P	0.440	0.240	-	0.420	0.220	2.52	1.40	6.72	2.80	18.5	441.4	441.4
McMullen, Rangan (1978)	A2	P	0.254	0.254	-	0.222	0.222	2.53	2.53	5.16	7.82	38.2	380.0	285.0
	A3	P	0.254	0.254	-	0.219	0.219	3.97	3.97	8.00	8.94	39.4	352.4	360.0
	A4	P	0.254	0.254	-	0.219	0.219	5.73	5.73	11.36	12.42	39.2	351.0	360.0
	B1r	P	0.178	0.356	-	0.146	0.324	1.43	1.43	2.85	3.87	36.3	360.0	285.0
	B2	P	0.178	0.356	-	0.146	0.324	2.53	2.53	5.07	7.19	39.6	380.0	285.0
	B3	P	0.178	0.356	-	0.143	0.321	3.97	3.97	8.00	8.60	38.6	352.4	360.0
	B4	P	0.178	0.356	-	0.143	0.321	5.73	5.73	11.36	11.76	38.5	351.0	360.0
Rasmussen, Baker (1995)	B30.1	P	0.160	0.275	-	0.120	0.235	5.15	7.72	15.44	8.73	41.7	620.0	665.0
	B30.2	P	0.160	0.275	-	0.120	0.235	5.15	7.72	15.44	8.73	38.2	638.0	669.0
	B30.3	P	0.160	0.275	-	0.120	0.235	5.15	7.72	15.44	8.73	36.3	605.0	672.0
	B50.1	P	0.160	0.275	-	0.120	0.235	5.15	7.72	15.44	8.73	61.8	612.0	665.0
	B50.2	P	0.160	0.275	-	0.120	0.235	5.15	7.72	15.44	8.73	57.1	614.0	665.0
	B50.3	P	0.160	0.275	-	0.120	0.235	5.15	7.72	15.44	8.73	61.7	612.0	665.0
	B70.1	P	0.160	0.275	-	0.120	0.235	5.15	7.72	15.44	8.73	77.3	617.0	658.0
	B70.2	P	0.160	0.275	-	0.120	0.235	5.15	7.72	15.44	8.73	76.9	614.0	656.0
	B70.3	P	0.160	0.275	-	0.120	0.235	5.15	7.72	15.44	8.73	76.2	617.0	663.0
	B110.1	P	0.160	0.275	-	0.120	0.235	5.09	7.63	15.44	8.73	109.8	618.0	655.0
	B110.2	P	0.160	0.275	-	0.120	0.235	5.09	7.63	15.44	8.73	105.0	634.0	660.0
** P—Plain section; H—Hollow section.														

**Ref.**	**Beam**	******	x	y	t	$x_{1}$	$y_{1}$	$A_{l1}$	$A_{l2}$	$A_{l}$	$A_{t}/s$	$f_{c}$	$f_{yl}$	$f_{yt}$
			**(m)**	**(m)**	**(m)**	**(m)**	**(m)**	**(cm^2^)**	**(cm^2^)**	**(cm^2^)**	**(cm^2^/m)**	**(MPa)**	**(MPa)**	**(MPa)**
	B110.3	P	0.160	0.275	-	0.120	0.235	5.15	7.72	15.44	8.73	105.1	629.0	655.0
Koutchoukali, Belarbi (2001)	B5UR1	P	0.203	0.305	-	0.165	0.267	2.53	2.53	5.16	6.56	39.6	386.0	373.0
	B7UR1	P	0.203	0.305	-	0.165	0.267	2.53	2.53	5.16	6.56	64.6	386.0	399.0
	B9UR1	P	0.203	0.305	-	0.165	0.267	2.53	2.53	5.16	6.56	75.0	386.0	373.0
	B12UR1	P	0.203	0.305	-	0.165	0.267	2.53	2.53	5.16	6.56	80.6	386.0	399.0
	B12UR2	P	0.203	0.305	-	0.165	0.267	2.53	2.53	5.16	6.95	76.2	386.0	386.0
	B12UR3	P	0.203	0.305	-	0.165	0.267	2.53	3.25	6.58	7.46	72.9	379.5	386.0
	B12UR4	P	0.203	0.305	-	0.165	0.267	2.53	3.80	7.74	7.88	75.9	373.0	386.0
	B12UR5	P	0.203	0.305	-	0.165	0.267	3.97	3.97	8.00	10.13	76.7	380.0	386.0
	B14UR1	P	0.203	0.305	-	0.165	0.267	2.53	2.53	5.16	6.56	93.9	386.0	386.0
Fang, Shiau (2004)	H-06-06	P	0.350	0.500	-	0.300	0.450	3.97	9.93	11.92	7.13	78.5	440.0	440.0
	H-06-12	P	0.350	0.500	-	0.300	0.450	5.07	12.67	20.65	7.10	78.5	410.0	440.0
	H-07-10	P	0.350	0.500	-	0.300	0.450	5.73	14.33	17.03	7.89	68.4	500.0	420.0
	H-07-16	P	0.350	0.500	-	0.300	0.450	8.60	22.92	28.39	7.89	68.4	500.0	420.0
	H-12-12	P	0.350	0.500	-	0.300	0.450	5.07	12.67	20.65	14.19	78.5	410.0	440.0
	H-12-16	P	0.350	0.500	-	0.300	0.450	8.60	18.62	28.39	14.19	78.5	520.0	440.0
	H-14-10	P	0.350	0.500	-	0.300	0.450	5.73	14.33	17.03	16.13	68.4	500.0	360.0
	H-20-20	P	0.350	0.500	-	0.300	0.450	8.60	22.92	34.06	23.46	78.5	560.0	440.0
	N-06-06	P	0.350	0.500	-	0.300	0.450	3.97	9.93	12.00	7.10	35.5	440.0	440.0
	N-06-12	P	0.350	0.500	-	0.300	0.450	5.07	12.67	20.65	7.10	35.5	410.0	440.0
	N-07-10	P	0.350	0.500	-	0.300	0.450	5.73	14.33	17.03	7.89	33.5	500.0	420.0
	N-07-16	P	0.350	0.500	-	0.300	0.450	8.60	20.06	28.39	7.89	33.5	500.0	420.0
	N-12-12	P	0.350	0.500	-	0.300	0.450	5.07	12.67	20.65	14.19	35.5	410.0	440.0
	N-12-16	P	0.350	0.500	-	0.300	0.450	8.60	20.06	28.39	14.19	35.5	520.0	440.0
	N-14-10	P	0.350	0.500	-	0.300	0.450	5.73	14.33	17.03	16.13	33.5	500.0	360.0
	N-20-20	P	0.350	0.500	-	0.300	0.450	8.60	22.92	34.06	23.46	35.5	560.0	440.0
Chiu et al. (2007)	HAS-51-50	P	0.420	0.420	-	0.370	0.370	3.80	3.25	9.03	5.94	76.0	396.0	385.0
	NAS-61-35	P	0.420	0.420	-	0.370	0.370	4.68	4.69	10.80	4.19	48.0	394.0	385.0
	HAS-90-50	P	0.420	0.420	-	0.370	0.370	5.96	5.96	15.89	5.94	78.0	400.0	385.0
	NBS-43-44	P	0.350	0.500	-	0.300	0.450	2.53	3.80	7.60	5.09	35.0	400.0	385.0
	HBS-74-17	P	0.350	0.500	-	0.300	0.450	5.73	6.44	12.89	2.02	67.0	505.0	600.0
	HBS-82-13	P	0.350	0.500	-	0.300	0.450	5.73	7.16	14.31	1.49	67.0	493.0	600.0
	NBS-82-13	P	0.350	0.500	-	0.300	0.450	5.73	7.16	14.31	1.49	35.0	493.0	600.0
	HBS-60-61	P	0.350	0.500	-	0.300	0.450	3.97	5.24	10.48	7.13	67.0	402.0	385.0
	HCS-52-50	P	0.250	0.700	-	0.200	0.650	2.53	4.51	9.03	5.09	76.0	396.0	385.0
	HCS-91-50	P	0.250	0.700	-	0.200	0.650	3.97	7.94	15.89	5.09	78.0	400.0	385.0
Lee and Kim (2010)	T1-1	P	0.300	0.350	-	0.260	0.310	2.53	2.53	5.07	5.48	43.2	410.0	370.0
	T1-2	P	0.300	0.350	-	0.260	0.310	2.53	3.80	7.60	8.39	44.0	410.0	370.0
	T1-3	P	0.300	0.350	-	0.260	0.310	2.53	5.07	10.14	10.97	41.7	410.0	370.0
	T1-4	P	0.300	0.350	-	0.260	0.310	3.97	5.96	11.92	16.89	42.6	510.0	355.0
	T2-2	P	0.300	0.350	-	0.260	0.310	3.97	5.96	7.94	5.48	41.7	510.0	370.0
** P—Plain section; H—Hollow section.														

**Ref.**	**Beam**	******	x	y	t	$x_{1}$	$y_{1}$	$A_{l1}$	$A_{l2}$	$A_{l}$	$A_{t}/s$	$f_{c}$	$f_{yl}$	$f_{yt}$
			**(m)**	**(m)**	**(m)**	**(m)**	**(m)**	**(cm^2^)**	**(cm^2^)**	**(cm^2^)**	**(cm^2^/m)**	**(MPa)**	**(MPa)**	**(MPa)**
	T2-3	P	0.300	0.350	-	0.260	0.310	3.97	5.96	11.92	8.10	42.7	510.0	370.0
	T2-4	P	0.300	0.350	-	0.260	0.310	5.73	7.00	14.00	9.51	42.6	512.4	370.0
Peng, Wong (2011)	SW12-1	P	0.150	1.200	-	0.100	1.150	2.26	11.31	22.62	3.93	44.2	480.0	459.0
	SW10-1	P	0.150	1.000	-	0.100	0.950	2.26	9.05	18.10	3.93	29.5	499.0	459.0
	SW10-2	P	0.150	1.000	-	0.098	0.948	2.26	9.05	18.10	7.54	44.2	480.0	480.0
	SW10-3	P	0.150	1.000	-	0.098	0.948	2.26	9.05	18.10	11.31	29.5	499.0	499.0
	SW10-4	P	0.150	1.000	-	0.094	0.944	4.02	16.08	32.16	16.08	33.8	497.0	497.0
	SW8-1	P	0.150	0.800	-	0.102	0.752	1.57	7.07	14.14	4.02	29.5	459.0	433.0
	SW8-2	P	0.150	0.800	-	0.098	0.748	1.57	7.07	14.14	11.31	29.5	459.0	499.0
Joh et al. (2019)	RA-SD4-3.2-0.3-3.28	P	0.300	0.400	-	0.270	0.370	11.91	11.91	30.97	7.13	73.7	452.0	484.0
	RA-SD5-3.2-0.3-3.21	P	0.300	0.400	-	0.270	0.370	11.61	11.61	30.97	6.48	73.7	499.0	538.0
	RA-SD6-3.2-0.2-3.21	P	0.300	0.400	-	0.270	0.370	11.61	11.61	30.97	6.48	73.7	630.0	538.0
	RA-SD4-3.2-0.5-2.13	P	0.300	0.400	-	0.270	0.370	5.73	8.60	17.19	7.13	84.7	456.0	484.0
	RA-SD5-3.2-0.7-1.63	P	0.300	0.400	-	0.270	0.370	3.97	5.96	11.92	6.48	84.7	529.0	538.0
	RA-SD6-3.2-0.6-1.63	P	0.300	0.400	-	0.270	0.370	3.97	5.96	11.92	6.48	84.7	627.0	538.0
	RA-SD4-3.2-1.1-1.33	P	0.300	0.400	-	0.270	0.370	2.53	3.80	7.60	7.13	83.1	474.0	484.0
	RA-SD5-3.2-1.0-1.26	P	0.300	0.400	-	0.270	0.370	2.53	3.80	7.60	6.48	83.1	522.0	538.0
	RA-SD6-3.2-0.8-1.26	P	0.300	0.400	-	0.270	0.370	3.97	3.97	7.94	6.48	83.1	627.0	538.0
Ju et al. (2019)	MR30-0.77	P	0.350	0.500	-	0.300	0.450	2.53	3.80	7.60	3.96	29.3	489.8	467.5
	MT30-1.32	P	0.350	0.500	-	0.300	0.450	5.73	8.60	17.19	3.96	29.3	500.4	467.5
	MT40-1.32	P	0.350	0.500	-	0.300	0.450	5.73	8.60	17.19	3.96	40.3	500.4	467.5
	MT40-1.89	P	0.350	0.500	-	0.300	0.450	5.73	8.60	17.19	10.57	40.3	489.8	489.8
Ibrahim et al. (2020)	NSC-S1-C30	P	0.200	0.300	-	0.168	0.268	2.26	2.26	4.52	6.28	42.1	689.7	534.1
	NSC-S1-C45	P	0.200	0.300	-	0.138	0.238	2.26	2.26	4.52	6.28	39.4	689.7	534.1
	HSC-C30	P	0.200	0.300	-	0.168	0.268	2.26	2.26	4.52	6.28	60.8	689.7	534.1
	HSC-C45	P	0.200	0.300	-	0.138	0.238	2.26	2.26	4.52	6.28	60.8	689.7	534.1
Kim et al. (2020)	S08-3-65	P	0.400	0.600	-	0.310	0.510	5.24	7.77	18.08	10.97	35.4	313.3	334.9
	S08-4-90	P	0.400	0.600	-	0.310	0.510	2.85	5.23	13.30	7.92	35.4	474.6	485.8
	S08-5-122.5	P	0.400	0.600	-	0.310	0.510	3.80	3.96	10.45	5.82	35.4	569.6	595.9
	S10-3-52.5	P	0.400	0.600	-	0.310	0.510	5.24	9.93	22.39	13.58	35.4	320.5	334.0
	S10-4-72.5	P	0.400	0.600	-	0.310	0.510	5.07	5.78	16.63	9.83	35.4	467.6	485.1
	S10-5-100	P	0.400	0.600	-	0.310	0.510	3.80	5.23	12.98	7.13	35.4	567.6	595.2
	S06-3-90	P	0.400	0.600	-	0.310	0.510	2.85	5.23	13.30	7.92	35.4	319.4	334.3
	S10-5-90	P	0.400	0.600	-	0.310	0.510	3.96	5.78	14.41	7.92	35.4	569.6	594.8
	S08-3-72.5	P	0.400	0.600	-	0.310	0.510	5.07	5.78	16.63	9.83	35.4	308.8	334.8
	S12-5-72.5	P	0.400	0.600	-	0.310	0.510	5.24	7.77	18.05	9.83	35.4	565.1	595.2
Hsu (1968)	D3	H	0.254	0.381	0.064	0.216	0.343	*	*	11.36	10.16	28.4	341.4	333.1
	D4	H	0.254	0.381	0.064	0.216	0.343	30.97	30.97	15.48	14.01	30.6	330.3	333.1
[17,38]	T0	H	0.500	0.500	0.080	0.430	0.430	*	*	32.16	10.28	45.1	345.2	357.0
	T1	H	0.500	0.500	0.080	0.454	0.454	4.52	4.52	18.10	10.28	35.3	356.7	356.7
	T2	H	0.500	0.500	0.080	0.430	0.430	*	*	18.10	10.28	35.3	357.0	357.0
* No sufficient data; ** P—Plain section; H—Hollow section.														

**Ref.**	**Beam**	******	x	y	t	$x_{1}$	$y_{1}$	$A_{l1}$	$A_{l2}$	$A_{l}$	$A_{t}/s$	$f_{c}$	$f_{yl}$	$f_{yt}$
			**(m)**	**(m)**	**(m)**	**(m)**	**(m)**	**(cm^2^)**	**(cm^2^)**	**(cm^2^)**	**(cm^2^/m)**	**(MPa)**	**(MPa)**	**(MPa)**
	T5	H	0.800	0.400	0.080	0.730	0.330	*	*	10.00	10.28	47.1	528.6	512.9
[19]	VH1	H	0.324	0.324	0.080	0.304	0.304	1.15	1.15	3.46	2.88	17.2	447.3	447.3
	VH2	H	0.324	0.324	0.080	0.304	0.304	*	*	6.91	5.76	17.2	447.3	447.3
Bernardo, Lopes (2009)	A1	H	0.600	0.600	0.098	0.537	0.547	*	*	6.53	3.14	48.4	695.9	636.7
	A2	H	0.600	0.600	0.107	0.538	0.531	4.62	4.62	13.95	6.28	47.3	672.4	695.9
	A3	H	0.600	0.600	0.109	0.535	0.535	5.65	5.65	18.10	8.27	46.2	672.4	714.8
	A4	H	0.600	0.600	0.104	0.520	0.525	7.95	7.95	23.75	11.22	54.8	723.9	714.8
	A5	H	0.600	0.600	0.104	0.528	0.528	9.68	9.68	30.66	14.14	53.1	723.9	672.4
	B2	H	0.600	0.600	0.108	0.533	0.534	4.78	4.78	14.58	6.70	69.8	672.4	695.9
	B3	H	0.600	0.600	0.109	0.535	0.537	7.95	7.95	23.75	11.22	77.8	723.9	714.8
	B4	H	0.600	0.600	0.112	0.523	0.536	10.05	10.05	32.17	15.08	79.8	723.9	672.4
	B5	H	0.600	0.600	0.117	0.518	0.518	12.06	12.06	40.21	18.85	76.4	723.9	672.4
	C1	H	0.600	0.600	0.097	0.540	0.549	*	*	6.53	3.14	91.7	695.9	636.7
	C2	H	0.600	0.600	0.100	0.532	0.533	4.62	4.62	13.95	6.28	94.8	672.4	695.9
	C3	H	0.600	0.600	0.103	0.545	0.540	7.95	7.95	23.75	10.47	91.6	723.9	714.8
	C4	H	0.600	0.600	0.103	0.546	0.545	9.68	9.68	30.66	14.14	91.4	723.9	672.4
	C5	H	0.600	0.600	0.104	0.540	0.543	12.32	12.32	36.69	17.40	96.7	723.9	672.4
	C6	H	0.600	0.600	0.104	0.533	0.529	14.07	14.07	48.25	22.62	87.5	723.9	672.4
Chiu et al. (2007)	HAH-81-35	H	0.420	0.420	0.075	0.370	0.370	5.73	6.44	14.31	4.19	78.0	493.0	385.0
	NCH-62-33	H	0.250	0.700	0.075	0.200	0.650	3.97	5.40	10.80	3.40	48.0	394.0	385.0
	HCH-91-42	H	0.250	0.700	0.075	0.200	0.650	3.97	7.94	15.89	4.32	78.0	400.0	385.0
Jeng (2014)	A095c	H	0.497	0.711	0.145	0.437	0.651	*	*	13.16	9.93	35.1	371.0	381.0
	A120a	H	0.502	0.719	0.184	0.442	0.659	*	*	20.00	7.59	27.6	464.0	380.0
	B065b	H	0.503	0.710	0.092	0.443	0.650	*	*	50.97	9.93	39.2	452.0	380.0
	B080a	H	0.500	0.721	0.112	0.440	0.661	*	*	28.39	12.90	46.5	454.0	392.0
	B110a	H	0.498	0.710	0.155	0.438	0.650	*	*	20.00	8.60	48.1	453.0	369.0
	C100a	H	0.499	0.723	0.127	0.439	0.663	*	*	28.39	12.90	90.6	466.0	447.0
	D075a	H	0.498	0.734	0.087	0.438	0.674	*	*	28.39	12.90	94.9	469.0	381.0
	D090a	H	0.501	0.722	0.105	0.441	0.662	*	*	28.39	12.90	105.7	466.0	447.0
Kim et al. (2020)	H08-3-65	H	0.400	0.600	0.100	0.310	0.510	5.24	7.77	18.08	10.97	36.5	361.1	352.2
	H08-4-90	H	0.400	0.600	0.100	0.310	0.510	3.96	5.23	13.30	7.92	36.5	445.7	448.9
	H08-5-100	H	0.400	0.600	0.100	0.310	0.510	3.25	5.23	11.88	7.13	36.5	545.5	539.8
	H10-3-52.5	H	0.400	0.600	0.100	0.310	0.510	5.96	9.21	22.39	13.58	36.5	356.9	352.4
	H10-4-72.5	H	0.400	0.600	0.100	0.310	0.510	5.07	5.78	16.63	9.83	36.5	444.5	447.9
	H10-5-80	H	0.400	0.600	0.100	0.310	0.510	3.96	5.78	14.41	8.91	36.5	546.3	538.6
	H06-3-90	H	0.400	0.600	0.100	0.310	0.510	2.85	5.23	13.30	7.92	36.5	359.1	351.0
	H10-5-135	H	0.400	0.600	0.100	0.310	0.510	3.25	3.8	9.02	5.28	36.5	548.1	540.3
	H08-3-72.5	H	0.400	0.600	0.100	0.310	0.510	5.07	5.78	16.63	9.83	36.5	359.4	352.7
	H12-5-72.5	H	0.400	0.600	0.100	0.310	0.510	5.07	5.78	16.63	9.83	36.5	404.1	538.7
* No sufficient data; ** P—Plain section; H—Hollow section.														

**Table A3:** Theoretical torsional strengths.

Ref.	Beam	**	$T_{R,exp}$	$T_{R,th}^{SiNiP18}$	$T_{R,th}^{ACI89}$	$T_{R,th}^{ACI19}$	$T_{R,th}^{MC90}$	$T_{R,th}^{MC10}$	$T_{R,th}^{EC2}$	$T_{R,th}^{CSA14}$	$T_{R,th}^{Rahal}\;$	$T_{R,th}^{Prop}\;$
			(kNm)	(kNm)	(kNm)	(kNm)	(kNm)	(kNm)	(kNm)	(kNm)	(kNm)	(kNm)
Hsu (1968)	B1	P	22.30	16.71	22.58	19.0	20.4	12.7	24.9	20.0	21.3	22.97
	B3	P	37.48	37.32	37.16	29.4	32.0	27.0	38.4	43.6	36.8	37.87
	B4 *	P	47.30	50.25	44.11	29.9	43.6	29.2	52.3	59.5	46.0	46.51
	B5 *	P	56.11	67.68	45.14	30.6	57.2	31.2	70.0	63.7	51.5	51.42
	B6 *	P	61.64	70.86	43.85	29.7	54.9	28.9	68.2	60.1	50.6	50.54
	B7	P	26.87	23.25	36.71	27.5	21.3	25.3	25.5	29.0	27.3	28.73
	B8	P	32.51	33.00	42.25	28.7	31.9	24.5	38.2	43.5	36.4	37.40
	B9	P	29.80	29.86	22.77	28.7	22.1	23.1	26.5	30.2	28.6	30.14
	B10	P	34.40	61.33	22.39	28.6	34.1	15.4	40.9	46.5	38.1	38.94
	C2	P	15.30	15.23	15.28	14.7	15.3	10.5	22.4	16.9	15.8	16.79
	C4 *	P	25.29	33.72	25.50	14.8	24.0	15.2	32.0	29.1	27.2	27.48
	C5 *	P	29.69	44.64	28.42	14.8	26.9	15.4	37.2	29.1	27.6	27.60
	C6 *	P	34.21	45.21	28.60	14.9	27.5	15.9	37.9	29.5	27.7	27.71
	G2	P	40.30	29.01	39.72	33.2	40.3	36.1	42.6	34.9	36.7	39.24
	G3	P	49.56	42.17	49.28	43.9	37.2	35.8	56.3	50.2	46.3	47.91
	G4	P	64.80	55.40	57.91	45.1	49.3	39.4	69.0	66.4	56.8	57.91
	G5 *	P	71.91	74.68	56.48	44.0	66.5	37.4	76.0	85.8	69.4	69.29
	G6	P	39.10	26.53	39.41	32.9	25.7	39.5	29.4	34.7	36.3	38.78
	G7	P	52.61	39.56	52.00	47.2	38.1	43.3	43.5	51.3	48.1	50.14
	G8	P	73.38	57.33	57.98	45.1	54.3	39.1	62.1	73.2	60.8	61.64
	I2	P	36.00	26.87	33.17	30.4	30.0	37.4	31.3	32.1	32.0	34.54
	I3	P	45.61	39.05	40.66	37.1	33.4	46.2	40.1	45.6	40.9	43.14
	I4	P	58.02	49.93	51.03	37.2	43.4	47.8	52.1	59.3	49.2	51.00
	I5	P	70.67	65.14	54.81	37.2	56.7	48.5	68.0	77.4	57.8	57.77
	I6 *	P	76.65	84.13	55.27	37.5	71.1	49.0	85.3	95.4	58.1	58.06
	J1	P	21.45	17.22	20.33	19.7	15.2	8.2	18.2	20.7	19.6	20.37
	J2 *	P	29.13	26.33	27.78	21.1	23.1	8.7	27.7	30.3	26.4	26.66
	J3 *	P	35.22	41.53	33.57	22.8	33.3	11.7	41.3	35.2	36.7	36.40
	J4 *	P	40.64	41.19	33.44	22.7	33.3	12.1	41.3	34.9	42.6	41.72
	K2	P	23.71	20.43	21.15	14.9	17.6	18.6	19.0	21.4	22.7	23.95
	K3 *	P	28.45	29.96	20.60	14.5	23.8	17.5	25.8	29.5	29.3	30.11
	K4 *	P	35.00	32.92	20.45	14.4	35.2	17.1	38.2	29.1	30.1	30.08
	M1	P	30.37	23.41	23.66	24.9	19.2	26.5	23.1	26.3	26.1	27.80
	M2	P	40.53	33.98	30.26	30.6	27.6	27.9	33.2	37.7	33.7	35.17
	M3	P	43.80	44.15	34.87	28.7	35.6	23.5	42.8	48.6	39.4	40.16
	M4 *	P	49.56	57.97	42.09	28.5	47.0	23.6	56.4	55.3	47.8	47.83
	M5 *	P	55.65	68.83	43.22	29.3	51.8	25.5	64.3	58.4	50.1	50.10
	M6 *	P	60.06	72.22	44.27	30.0	52.8	26.3	65.5	61.2	50.9	50.82
	N1	P	9.09	6.62	8.63	7.5	5.9	8.5	6.7	7.9	7.9	8.72
	N1a	P	8.99	6.58	8.65	7.5	5.8	8.2	6.7	7.9	7.9	8.65

* Fragile failure; ** P—Plain section; H—Hollow section.

**Ref.**	**Beam**	******	$T_{R,exp}$	$T_{R,th}^{SiNiP18}$	$T_{R,th}^{ACI89}$	$T_{R,th}^{ACI19}$	$T_{R,th}^{MC90}$	$T_{R,th}^{MC10}$	$T_{R,th}^{EC2}$	$T_{R,th}^{CSA14}$	$T_{R,th}^{Rahal}$	$T_{R,th}^{Prop}$
			**(kNm)**	**(kNm)**	**(kNm)**	**(kNm)**	**(kNm)**	**(kNm)**	**(kNm)**	**(kNm)**	**(kNm)**	**(kNm)**
	N2	P	14.45	11.37	12.97	10.2	10.3	9.3	11.7	13.9	11.8	12.54
	N2a	P	13.21	11.63	12.54	7.6	5.9	9.1	6.7	8.0	7.9	8.66
	N3	P	12.19	9.33	11.53	9.7	8.8	7.6	10.0	11.9	10.4	11.08
	N4 *	P	15.69	15.04	12.29	9.7	13.5	8.2	15.5	18.3	14.1	14.60
[17]	T4	P	138.61	80.95	124.21	126.6	83.8	121.6	111.7	133.4	124.7	128.92
Leonhardt, Schelling (1974)	VB2 *	P	42.11	24.55	53.57	38.7	34.4	17.3	39.8	50.7	39.6	40.73
	VB3	P	46.40	24.55	57.01	47.1	34.4	29.0	39.8	50.7	42.2	44.37
	VB4	P	48.54	24.55	59.50	48.1	34.4	35.3	39.8	50.7	43.9	46.77
	VM1	P	13.89	8.58	16.66	12.5	9.0	10.2	10.4	13.1	11.2	12.23
	VM2	P	39.17	26.14	46.25	35.7	25.5	29.9	29.5	37.6	33.8	36.08
	VM3	P	100.80	70.62	113.58	92.8	66.1	76.4	76.5	97.7	88.0	92.02
	VQ1	P	21.11	15.99	24.54	25.1	15.8	12.3	21.1	26.4	23.3	24.48
	VQ3	P	19.98	9.62	35.85	20.5	15.1	9.0	16.4	21.6	21.7	22.95
	VQ9	P	21.90	11.08	44.31	19.9	14.9	10.6	15.6	21.0	24.2	25.64
	VS2-VQ2	P	19.53	11.74	29.19	20.1	14.3	10.4	16.6	21.2	20.4	21.74
	VS3	P	28.56	16.07	37.12	30.0	21.5	10.8	24.8	31.6	27.2	28.25
	VS4-VQ5 *	P	34.32	20.53	44.92	32.8	28.7	10.4	33.2	42.3	33.1	33.79
	VS9 *	P	21.56	16.31	32.69	25.2	18.0	9.1	20.8	26.5	23.6	24.70
	VS10-VB1 *	P	33.30	25.70	52.94	32.8	36.0	10.4	41.7	53.1	38.8	39.03
	VU1	P	23.93	13.75	43.11	26.6	19.0	11.4	22.0	28.0	24.9	26.15
	VU2 *	P	30.37	16.47	43.11	32.6	23.3	11.2	26.9	34.3	28.7	29.75
	VU3 *	P	31.04	17.77	35.28	32.4	23.2	9.4	26.9	34.2	28.5	29.36
	VU4	P	25.96	16.39	28.02	26.6	19.0	8.4	22.0	28.0	24.7	25.85
McMullen, Rangan (1978)	A2	P	22.58	17.01	17.62	18.6	13.0	21.6	17.4	19.6	18.1	19.43
	A3	P	27.77	24.69	22.16	18.7	18.8	23.6	25.1	27.7	23.4	24.68
	A4 *	P	34.43	35.16	28.10	18.6	26.3	23.8	35.1	38.8	29.6	30.55
	B1r	P	12.30	8.08	11.79	8.8	12.3	9.9	10.8	9.3	11.0	12.29
	B2	P	20.80	15.05	18.22	16.5	20.8	16.5	17.7	17.4	17.2	18.62
	B3	P	25.29	22.23	23.21	15.9	19.8	21.6	22.6	25.2	22.6	23.78
	B4 *	P	31.72	31.27	23.17	15.9	26.7	21.6	30.5	35.1	28.0	28.00
Rasmussen, Baker (1995)	B30.1 *	P	16.62	29.36	15.11	8.2	21.2	11.1	25.7	19.9	17.0	17.04
	B30.2 *	P	15.29	26.89	14.46	7.8	19.6	10.3	23.8	18.2	16.6	16.59
	B30.3 *	P	15.25	25.56	14.09	7.6	19.1	9.8	23.1	17.3	16.4	16.34
	B50.1 *	P	19.95	43.51	18.39	9.9	28.6	15.3	34.8	29.4	19.2	19.17
	B50.2 *	P	18.46	40.20	17.68	9.6	27.0	14.3	32.9	27.2	18.7	18.72
	B50.3 *	P	19.13	43.44	18.37	9.9	28.6	15.2	34.8	29.4	19.2	19.16
	B70.1 *	P	20.06	49.42	20.57	11.1	31.2	17.9	36.5	36.8	20.5	20.50
	B70.2 *	P	20.74	49.19	20.51	11.1	31.1	17.9	36.4	36.6	20.5	20.47
	B70.3 *	P	20.96	49.46	20.42	11.0	31.3	17.8	36.7	36.3	20.4	20.41
	B110.1 *	P	24.72	48.95	24.51	13.3	31.2	23.1	36.5	44.4	22.8	22.78
	B110.2 *	P	23.62	50.12	23.97	13.0	31.7	22.3	37.1	45.2	22.5	22.47
* Fragile failure; ** P—Plain section; H—Hollow section.												

**Ref.**	**Beam**	******	$T_{R,exp}$	$T_{R,th}^{SiNiP18}$	$T_{R,th}^{ACI89}$	$T_{R,th}^{ACI19}$	$T_{R,th}^{MC90}$	$T_{R,th}^{MC10}$	$T_{R,th}^{EC2}$	$T_{R,th}^{CSA14}$	$T_{R,th}^{Rahal}$	$T_{R,th}^{Prop}$
			**(kNm)**	**(kNm)**	**(kNm)**	**(kNm)**	**(kNm)**	**(kNm)**	**(kNm)**	**(kNm)**	**(kNm)**	**(kNm)**
	B110.3 *	P	24.77	50.24	23.98	13.0	31.5	22.3	36.8	44.8	22.5	22.48
Koutchoukali, Belarbi (2001)	B5UR1	P	19.40	16.42	18.14	16.0	14.5	22.4	17.4	18.8	18.1	19.46
	B7UR1	P	18.90	16.87	20.50	18.4	15.0	25.7	18.0	19.4	20.0	22.11
	B9UR1	P	21.10	16.42	20.12	17.8	14.5	24.8	17.4	18.8	20.0	22.36
	B12UR1	P	19.40	16.87	21.28	18.4	15.0	25.7	18.0	19.4	20.8	23.21
	B12UR2	P	18.40	17.04	21.41	18.6	15.2	26.0	18.2	19.6	20.8	23.10
	B12UR3	P	22.50	19.10	22.29	21.6	17.6	30.1	21.2	22.8	22.9	25.15
	B12UR4	P	23.70	19.09	23.28	22.1	19.5	33.3	23.4	25.2	24.7	27.03
	B12UR5	P	24.00	25.70	27.89	22.2	22.2	37.7	26.6	29.3	27.5	29.83
	B14UR1	P	21.00	16.64	21.43	18.1	14.8	25.2	17.7	19.1	21.0	23.74
Fang, Shiau (2004)	H-06-06	P	92.00	57.90	85.00	76.0	57.8	113.1	70.1	80.1	87.4	95.25
	H-06-12	P	115.10	66.14	84.79	96.4	73.3	135.1	88.9	101.6	103.1	110.75
	H-07-10	P	126.70	86.66	85.35	99.6	75.8	128.9	91.8	104.9	103.2	109.71
	H-07-16	P	144.50	122.53	85.35	113.5	97.8	109.1	118.5	135.5	123.4	129.06
	H-12-12	P	155.30	80.31	133.52	121.6	103.7	158.3	125.7	143.6	131.5	138.06
	H-12-16	P	196.00	140.15	133.52	121.6	137.0	146.3	166.0	189.7	159.7	164.77
	H-14-10	P	135.20	97.96	124.21	113.5	100.3	149.9	121.5	138.9	125.6	131.16
	H-20-20 *	P	239.00	167.22	180.32	121.6	200.1	158.5	242.5	277.1	166.9	166.74
	N-06-06	P	79.70	57.84	72.98	76.1	57.9	80.6	70.2	80.2	77.0	80.17
	N-06-12	P	95.20	66.14	72.98	81.8	73.3	70.1	88.9	101.6	90.8	93.16
	N-07-10	P	111.70	86.66	75.24	79.4	75.8	68.7	91.8	104.9	92.1	93.90
	N-07-16	P	117.30	122.53	75.24	79.4	97.8	58.2	118.5	135.5	110.1	110.46
	N-12-12 *	P	116.80	80.31	121.26	81.8	103.7	82.1	125.7	143.6	115.8	116.13
	N-12-16 *	P	138.00	140.15	121.26	81.8	137.0	75.9	166.0	183.3	131.5	131.41
	N-14-10 *	P	125.00	97.96	114.11	79.4	100.3	79.9	121.5	138.9	112.0	112.26
	N-20-20 *	P	158.00	167.22	121.26	81.8	155.4	82.2	195.1	183.3	131.5	131.41
Chiu et al. (2007)	HAS-51-50	P	84.90	40.48	73.94	54.7	37.9	82.9	50.6	57.7	68.6	76.34
	NAS-61-35	P	74.70	48.22	56.00	50.2	34.8	75.0	46.3	52.8	60.0	65.34
	HAS-90-50	P	104.23	63.51	74.50	73.0	50.6	109.1	67.4	76.9	84.2	92.19
	NBS-43-44	P	60.60	34.20	54.67	45.8	34.8	69.1	42.2	48.2	53.8	57.82
	HBS-74-17	P	62.20	65.76	52.24	52.7	40.1	78.5	48.6	55.5	65.9	72.85
	HBS-82-13	P	56.30	68.29	47.26	47.1	35.8	70.1	43.4	49.6	60.9	67.83
	NBS-82-13	P	52.90	68.29	38.02	47.1	35.8	47.5	43.4	49.6	54.9	58.88
	HBS-60-61	P	93.70	52.35	76.14	63.7	48.5	94.8	58.8	67.2	75.3	82.25
	HCS-52-50	P	73.54	40.26	63.60	44.9	37.4	65.8	41.1	47.3	64.5	72.11
	HCS-91-50	P	95.86	59.72	63.93	59.9	49.9	86.1	54.8	63.1	79.2	87.07
Lee and Kim (2010)	T1-1	P	32.90	23.41	30.99	26.4	19.3	38.6	24.5	27.8	29.6	32.32
	T1-2	P	45.90	27.93	40.26	39.9	29.2	50.6	37.1	42.1	39.7	42.27
	T1-3	P	54.10	30.94	47.99	41.6	38.5	48.2	49.1	55.6	47.8	49.85
	T1-4	P	62.40	54.32	64.60	42.0	56.7	51.1	72.1	81.7	62.8	63.97
	T2-2	P	38.10	41.82	30.76	36.8	26.9	42.2	34.2	38.8	37.2	39.67
* Fragile failure; ** P—Plain section; H—Hollow section.												

**Ref.**	**Beam**	******	$T_{R,exp}$	$T_{R,th}^{SiNiP18}$	$T_{R,th}^{ACI89}$	$T_{R,th}^{ACI19}$	$T_{R,th}^{MC90}$	$T_{R,th}^{MC10}$	$T_{R,th}^{EC2}$	$T_{R,th}^{CSA14}$	$T_{R,th}^{Rahal}$	$T_{R,th}^{Prop}$
			**(kNm)**	**(kNm)**	**(kNm)**	**(kNm)**	**(kNm)**	**(kNm)**	**(kNm)**	**(kNm)**	**(kNm)**	**(kNm)**
	T2-3	P	50.20	44.88	39.14	42.1	40.1	42.8	51.0	57.7	49.3	51.36
	T2-4	P	56.40	62.46	43.55	42.0	47.1	43.4	60.0	68.0	55.2	56.94
Peng, Wong (2011)	SW12-1	P	74.00	66.82	43.22	39.7	48.7	59.0	50.3	57.8	87.3	91.40
	SW10-1	P	50.70	54.79	33.97	26.4	39.8	30.7	41.3	47.5	63.9	65.62
	SW10-2	P	68.00	58.02	49.70	31.0	52.2	59.0	54.3	64.5	76.3	76.21
	SW10-3	P	74.30	62.29	40.61	25.3	66.5	39.8	69.1	51.7	67.6	67.51
	SW10-4 *	P	80.50	76.05	43.46	24.9	87.1	44.2	91.6	54.5	70.4	70.32
	SW8-1	P	40.40	30.61	26.43	21.1	30.8	24.6	32.3	35.3	44.8	46.59
	SW8-2	P	60.10	34.63	32.48	19.5	50.2	31.7	52.7	39.8	52.3	52.30
Joh et al. (2019)	RA-SD4-3.2-0.3-3.28 *	P	86.80	97.32	58.91	75.6	74.0	60.9	91.1	110.0	86.5	90.30
	RA-SD5-3.2-0.3-3.21 *	P	88.00	103.65	59.32	75.6	78.2	58.9	96.2	116.2	89.8	93.41
	RA-SD6-3.2-0.2-3.21 *	P	89.40	126.46	59.32	75.6	87.8	53.7	108.1	130.5	93.5	93.41
	RA-SD4-3.2-0.5-2.13 *	P	76.30	63.56	60.39	78.1	55.4	80.1	68.1	82.3	72.2	77.41
	RA-SD5-3.2-0.7-1.63 *	P	74.50	53.42	60.80	70.4	49.9	82.7	61.4	74.2	67.1	72.47
	RA-SD6-3.2-0.6-1.63 *	P	70.00	61.20	60.80	76.7	54.3	79.0	66.9	80.8	71.2	76.49
	RA-SD4-3.2-1.1-1.33 *	P	74.20	35.28	60.18	53.0	37.5	78.8	46.2	55.8	54.8	60.21
	RA-SD5-3.2-1.0-1.26 *	P	67.70	37.83	60.59	55.9	39.6	83.1	48.7	58.9	56.9	62.29
	RA-SD6-3.2-0.8-1.26 *	P	69.90	54.08	60.59	62.6	44.4	86.2	54.6	66.0	61.6	66.96
Ju et al. (2019)	MR30-0.77	P	55.00	39.38	50.92	49.2	37.4	55.3	45.4	51.9	55.0	58.27
	MT30-1.32 *	P	57.00	80.08	50.92	74.3	56.9	43.7	69.0	78.8	73.8	76.05
	MT40-1.32 *	P	58.30	80.08	54.73	74.8	56.9	64.7	69.0	78.8	77.6	81.52
	MT40-1.89	P	98.40	93.65	106.57	87.1	94.1	89.9	114.1	130.4	110.4	112.27
Ibrahim et al. (2020)	NSC-S1-C30 *	P	19.70	24.21	23.08	17.0	20.8	20.9	24.9	27.9	23.1	24.34
	NSC-S1-C45 *	P	12.50	24.21	18.54	10.2	16.9	21.1	20.3	21.9	22.9	23.99
	HSC-C30 *	P	19.90	24.21	24.13	20.5	20.8	28.0	24.9	27.9	24.5	26.37
	HSC-C45 *	P	13.80	24.21	19.75	12.6	16.9	28.7	20.3	21.9	24.5	26.37
Kim et al. (2020)	S08-3-65	P	123.00	71.29	107.84	95.8	82.9	143.1	99.4	100.9	114.5	118.81
	S08-4-90	P	124.00	63.67	111.18	102.4	89.6	149.3	107.5	109.0	120.9	124.81
	S08-5-122.5	P	89.00	80.78	103.94	95.4	82.5	142.5	99.1	100.5	114.2	118.51
	S10-3-52.5	P	126.00	77.60	124.27	102.4	103.7	150.3	124.4	126.2	133.9	136.98
	S10-4-72.5 *	P	109.00	99.74	128.72	102.4	110.7	149.5	132.8	134.7	140.2	142.79
	S10-5-100 *	P	108.00	90.17	118.70	102.4	101.6	147.7	121.9	123.7	132.1	135.24
	S06-3-90	P	101.00	43.20	88.35	70.4	60.9	105.2	73.1	74.2	92.3	97.71
	S10-5-90 *	P	106.00	95.94	127.61	102.4	113.0	147.6	135.6	137.6	142.3	144.70
	S08-3-72.5	P	106.00	66.73	100.60	86.3	74.7	129.0	89.7	91.0	106.5	111.23
	S12-5-72.5 *	P	120.00	123.96	149.33	102.4	140.4	147.6	168.5	170.9	165.6	166.13
Hsu (1968)	D3	H	39.11	66.5	27.11	28.3	26.2	23.6	31.5	35.8	32.1	38.97
	D4 *	H	47.93	75.27	45.20	29.4	44.9	29.6	53.9	61.2	47.3	47.92
[17,38]	T0	H	185.50	116.4	91.25	112.2	99.9	139.4	133.3	146.6	146.8	161.65
	T1	H	140.01	70.87	106.45	110.6	83.8	132.0	111.7	133.4	124.7	128.92
	T2	H	143.10	74.0	87.13	99.3	76.2	127.2	101.6	111.8	116.7	128.99
* Fragile failure. ** P—Plain section; H—Hollow section.												

**Ref.**	**Beam**	******	$T_{R,exp}$	$T_{R,th}^{SiNiP18}$	$T_{R,th}^{ACI89}$	$T_{R,th}^{ACI19}$	$T_{R,th}^{MC90}$	$T_{R,th}^{MC10}$	$T_{R,th}^{EC2}$	$T_{R,th}^{CSA14}$	$T_{R,th}^{Rahal}$	$T_{R,th}^{Prop}$
			**(kNm)**	**(kNm)**	**(kNm)**	**(kNm)**	**(kNm)**	**(kNm)**	**(kNm)**	**(kNm)**	**(kNm)**	**(kNm)**
	T5	H	156.88	87.7	192.68	135.1	109.3	216.9	124.9	142.3	165.6	185.69
[19]	VH1	H	21.79	12.84	21.04	20.1	12.7	14.5	16.9	21.2	19.6	20.82
	VH2 *	H	34.50	25.6	46.24	32.9	30.2	15.5	41.6	51.2	49.9	32.35
Bernardo, Lopes (2009)	A1	H	150.79	108.5	122.50	101.7	67.9	150.9	90.6	107.1	134.7	147.43
	A2	H	254.08	145.39	193.85	212.2	144.6	313.0	192.8	223.6	227.8	236.84
	A3	H	299.92	185.31	239.37	239.4	192.6	317.6	256.7	298.2	277.2	281.58
	A4	H	368.22	269.46	291.30	238.1	266.6	352.9	355.5	398.5	357.8	359.54
	A5 *	H	412.24	324.19	334.84	238.9	328.7	338.0	438.3	499.1	412.2	408.84
	B2	H	273.28	152.10	216.87	223.3	152.7	329.1	203.6	235.3	251.7	266.80
	B3	H	355.85	269.46	321.28	312.1	266.6	471.8	355.5	414.1	378.5	388.14
	B4 *	H	437.85	339.83	374.47	317.1	347.8	480.9	463.7	529.4	457.6	463.09
	B5 *	H	456.19	413.87	434.23	309.5	434.7	474.4	579.6	641.3	496.1	495.73
	C1	H	151.76	109.4	146.65	102.5	67.9	151.5	90.6	108.0	149.2	169.46
	C2	H	266.14	145.39	215.62	211.0	144.6	319.0	192.8	222.4	254.5	275.56
	C3	H	351.17	263.00	314.00	326.1	257.6	508.0	343.5	407.3	379.2	393.43
	C4 *	H	450.31	324.19	374.22	330.7	328.7	506.0	438.3	524.1	449.6	460.21
	C5 *	H	467.26	407.83	436.52	338.1	398.9	532.7	531.9	629.1	519.5	526.94
	C6 *	H	521.33	487.91	464.52	309.2	521.6	494.1	695.5	798.7	516.7	516.31
Chiu et al. (2007)	HAH-81-35	H	94.31	68.82	52.95	64.6	44.8	96.7	59.7	68.1	77.4	85.31
	NCH-62-33	H	64.14	39.94	45.64	40.0	33.3	57.7	36.6	42.1	55.2	60.60
	HCH-91-42	H	87.51	56.61	58.00	55.0	45.8	79.4	50.3	58.0	74.6	82.64
Jeng (2014)	A095c	H	209.98	184.5	142.52	108.4	79.9	137.5	96.8	114.3	134.4	168.97
	A120a	H	215.25	189.8	96.76	101.6	65.4	112.1	79.3	107.0	124.7	184.33
	B065b	H	278.00	252.2	128.27	187.4	174.4	151.5	211.9	250.3	237.8	286.31
	B080a	H	300.66	275.2	243.23	250.9	198.2	309.1	239.8	283.5	267.9	273.75
	B110a	H	237.48	212.0	127.34	117.3	83.7	143.6	101.6	123.6	149.4	208.45
	C100a	H	370.15	344.7	308.37	291.2	214.7	393.6	259.4	306.8	315.3	333.10
	D075a	H	339.48	314.0	251.86	272.3	201.1	418.3	242.2	287.0	305.0	324.75
	D090a	H	343.08	317.6	298.37	292.0	214.9	424.1	260.1	307.6	324.0	345.40
Kim et al. (2020)	H08-3-65	H	128.00	79.94	112.05	104.0	91.2	146.4	109.5	111.1	123.1	127.15
	H08-4-90	H	130.00	74.29	106.20	96.4	83.4	146.8	100.1	101.6	115.6	120.10
	H08-5-100	H	117.00	76.27	111.77	104.0	90.7	146.5	108.9	110.5	122.6	126.70
	H10-3-52.5	H	143.00	92.53	129.59	104.0	112.4	146.7	134.8	136.8	142.4	145.15
	H10-4-72.5	H	127.00	94.04	122.35	104.0	103.7	146.6	124.4	126.2	134.6	137.91
	H10-5-80	H	125.00	93.70	129.86	104.0	111.7	147.2	134.0	136.0	141.8	144.59
	H06-3-90	H	102.00	47.39	91.44	76.5	66.2	122.2	79.5	80.6	98.3	103.69
	H10-5-135	H	95.00	70.56	92.83	78.9	68.2	126.0	81.9	83.1	100.4	105.70
	H08-3-72.5	H	114.00	75.47	104.53	95.6	82.7	145.9	99.3	100.7	114.9	119.46
	H12-5-72.5	H	129.00	93.25	139.33	104.0	108.4	152.7	130.1	132.0	138.9	141.88
* Fragile failure. ** P—Plain section; H—Hollow section.												

**Table A4:** Ratios of experimental to theoretical torsional strengths.

Ref.	Beam	**	$\frac{T_{R,exp}}{T_{R,th}^{SiNiP18}}$	$\frac{T_{R,exp}}{T_{R,th}^{ACI89}}$	$\frac{T_{R,exp}}{T_{R,th}^{ACI19}}$	$\frac{T_{R,exp}}{T_{R,th}^{MC90}}$	$\frac{T_{R,exp}}{T_{R,th}^{MC10}}$	$\frac{T_{R,exp}}{T_{R,th}^{EC2}}$	$\frac{T_{R,exp}}{T_{R,th}^{CSA14}}$	$\frac{T_{R,exp}}{T_{R,th}^{Rahal}}\;$	$\frac{T_{R,exp}}{T_{R,th}^{Prop}}\;$
Hsu (1968)	B1	P	1.33	0.99	1.17	1.09	1.76	0.90	1.11	1.05	0.97
	B3	P	1.00	1.01	1.28	1.17	1.39	0.98	0.86	1.02	0.99
	B4	P	0.94	1.07	1.58	1.09	1.62	0.90	0.80	1.03	1.02
	B5	P	0.83	1.24	1.83	0.98	1.80	0.80	0.88	1.09	1.09
	B6	P	0.87	1.41	2.07	1.12	2.13	0.90	1.03	1.22	1.22
	B7	P	1.16	0.73	0.98	1.26	1.06	1.05	0.93	0.98	0.94
	B8	P	0.99	0.77	1.13	1.02	1.33	0.85	0.75	0.89	0.87
	B9	P	1.00	1.31	1.04	1.35	1.29	1.12	0.99	1.04	0.99
	B10	P	0.56	1.54	1.20	1.01	2.24	0.84	0.74	0.90	0.88
	C2	P	1.00	1.00	1.04	1.00	1.46	0.68	0.90	0.97	0.91
	C4	P	0.75	0.99	1.71	1.05	1.67	0.79	0.87	0.93	0.92
	C5	P	0.67	1.04	2.00	1.10	1.93	0.80	1.02	1.07	1.08
	C6	P	0.76	1.20	2.29	1.24	2.15	0.90	1.16	1.23	1.23
	G2	P	1.39	1.01	1.22	1.00	1.12	1.16	1.15	1.10	1.03
	G3	P	1.18	1.01	1.13	1.33	1.38	1.15	0.99	1.07	1.03
	G4	P	1.17	1.12	1.44	1.32	1.64	0.58	0.98	1.14	1.12
	G5	P	0.96	1.27	1.64	1.08	1.92	0.95	0.84	1.04	1.04
	G6	P	1.47	0.99	1.19	1.52	0.99	1.33	1.13	1.08	1.01
	G7	P	1.33	1.01	1.12	1.38	1.22	1.21	1.03	1.09	1.05
	G8	P	1.28	1.27	1.63	1.35	1.88	1.18	1.00	1.21	1.19
	I2	P	1.34	1.09	1.18	1.20	0.96	1.15	1.12	1.13	1.04
	I3	P	1.17	1.12	1.23	1.36	0.99	1.14	1.00	1.12	1.06
	I4	P	1.16	1.14	1.56	1.34	1.21	1.11	0.98	1.18	1.14
	I5	P	1.08	1.29	1.90	1.25	1.46	1.04	0.91	1.22	1.22
	I6	P	0.91	1.39	2.04	1.08	1.57	0.90	0.80	1.32	1.32
	J1	P	1.25	1.06	1.09	1.41	2.62	1.18	1.04	1.09	1.05
	J2	P	1.11	1.05	1.38	1.26	3.36	1.05	0.96	1.11	1.09
	J3	P	0.85	1.05	1.55	1.06	3.01	0.85	1.00	0.96	0.97
	J4	P	0.99	1.22	1.79	1.22	3.36	0.98	1.16	0.95	0.97
	K2	P	1.16	1.12	1.59	1.35	1.27	1.25	1.11	1.04	0.99
	K3	P	0.95	1.38	1.96	1.20	1.63	1.10	0.97	0.97	0.94
	K4	P	1.06	1.71	2.42	0.99	2.05	0.92	1.20	1.16	1.16
	M1	P	1.30	1.28	1.22	1.58	1.14	1.31	1.16	1.17	1.09
	M2	P	1.19	1.34	1.32	1.47	1.45	1.22	1.07	1.20	1.15
	M3	P	0.99	1.26	1.53	1.23	1.86	1.02	0.90	1.11	1.09
	M4	P	0.85	1.18	1.74	1.05	2.10	0.88	0.90	1.04	1.04
	M5	P	0.81	1.29	1.90	1.07	2.19	0.87	0.95	1.11	1.11
	M6	P	0.83	1.36	2.00	1.14	2.28	0.92	0.98	1.18	1.18
	N1	P	1.37	1.05	1.20	1.55	1.07	1.36	1.14	1.15	1.04
	N1a	P	1.37	1.04	1.20	1.54	1.10	1.35	1.13	1.14	1.04
	N2	P	1.27	1.11	1.41	1.41	1.56	1.23	1.04	1.23	1.15

** P—Plain section; H—Hollow section.

**Ref.**	**Beam**	******	$\frac{T_{R,exp}}{T_{R,th}^{SiNiP18}}$	$\frac{T_{R,exp}}{T_{R,th}^{ACI89}}$	$\frac{T_{R,exp}}{T_{R,th}^{ACI19}}$	$\frac{T_{R,exp}}{T_{R,th}^{MC90}}$	$\frac{T_{R,exp}}{T_{R,th}^{MC10}}$	$\frac{T_{R,exp}}{T_{R,th}^{EC2}}$	$\frac{T_{R,exp}}{T_{R,th}^{CSA14}}$	$\frac{T_{R,exp}}{T_{R,th}^{Rahal}}$	$\frac{T_{R,exp}}{T_{R,th}^{Prop}}$
	N2a	P	1.14	1.05	1.75	2.25	1.45	1.97	1.66	1.67	1.52
	N3	P	1.31	1.06	1.26	1.39	1.60	1.22	1.03	1.17	1.10
	N4	P	1.04	1.28	1.62	1.16	1.91	1.02	0.86	1.12	1.07
[17]	T4	P	1.71	1.12	1.10	1.65	1.14	1.24	1.04	1.11	1.08
Leonhardt, Schelling (1974)	VB2	P	1.72	0.79	1.09	1.23	2.43	1.06	0.83	1.06	1.03
	VB3	P	1.89	0.81	0.98	1.35	1.60	1.17	0.92	1.10	1.05
	VB4	P	1.98	0.82	1.01	1.41	1.38	1.22	0.96	1.11	1.04
	VM1	P	1.62	0.83	1.11	1.55	1.36	1.34	1.06	1.24	1.14
	VM2	P	1.50	0.85	1.10	1.54	1.31	1.33	1.04	1.16	1.09
	VM3	P	1.43	0.89	1.09	1.53	1.32	1.32	1.03	1.14	1.10
	VQ1	P	1.32	0.86	0.84	1.34	1.72	1.00	0.80	0.91	0.86
	VQ3	P	2.08	0.56	0.98	1.32	2.23	1.21	0.93	0.92	0.87
	VQ9	P	1.98	0.49	1.10	1.47	2.07	1.41	1.04	0.90	0.85
	VS2-VQ2	P	1.66	0.67	0.97	1.36	1.88	1.18	0.92	0.96	0.90
	VS3	P	1.78	0.77	0.95	1.33	2.64	1.15	0.90	1.05	1.01
	VS4-VQ5	P	1.67	0.76	1.04	1.20	3.31	1.03	0.81	1.04	1.02
	VS9	P	1.32	0.66	0.86	1.20	2.38	1.04	0.81	0.91	0.87
	VS10-VB1	P	1.30	0.63	1.01	0.93	3.21	0.80	0.63	0.86	0.85
	VU1	P	1.74	0.56	0.90	1.26	2.09	1.09	0.85	0.96	0.92
	VU2	P	1.84	0.70	0.93	1.31	2.70	1.13	0.88	1.06	1.02
	VU3	P	1.75	0.88	0.96	1.34	3.32	1.16	0.91	1.09	1.06
	VU4	P	1.58	0.93	0.98	1.37	3.08	1.18	0.93	1.05	1.00
McMullen, Rangan (1978)	A2	P	1.33	1.28	1.21	1.73	1.05	1.30	1.15	1.25	1.16
	A3	P	1.12	1.25	1.49	1.48	1.17	1.11	1.00	1.19	1.13
	A4	P	0.98	1.23	1.85	1.31	1.45	0.98	0.89	1.16	1.13
	B1r	P	1.52	1.04	1.39	1.00	1.24	1.24	1.32	1.12	1.00
	B2	P	1.38	1.14	1.26	1.00	1.26	1.26	1.20	1.21	1.12
	B3	P	1.14	1.09	1.59	1.28	1.17	1.12	1.00	1.12	1.06
	B4	P	1.01	1.37	2.00	1.19	1.47	1.04	0.90	1.13	1.13
Rasmussen, Baker (1995)	B30.1	P	0.57	1.10	2.03	0.78	1.49	0.65	0.84	0.97	0.98
	B30.2	P	0.57	1.06	1.96	0.78	1.49	0.64	0.84	0.92	0.92
	B30.3	P	0.60	1.08	2.00	0.80	1.55	0.66	0.88	0.93	0.93
	B50.1	P	0.46	1.08	2.01	0.70	1.31	0.57	0.68	1.04	1.04
	B50.2	P	0.46	1.04	1.93	0.68	1.29	0.56	0.68	0.99	0.99
	B50.3	P	0.44	1.04	1.92	0.67	1.26	0.55	0.65	1.00	1.00
	B70.1	P	0.41	0.98	1.80	0.64	1.12	0.55	0.55	0.98	0.98
	B70.2	P	0.42	1.01	1.87	0.67	1.16	0.57	0.57	1.01	1.01
	B70.3	P	0.42	1.03	1.90	0.67	1.18	0.57	0.58	1.03	1.03
	B110.1	P	0.50	1.01	1.86	0.79	1.07	0.68	0.56	1.08	1.09
	B110.2	P	0.47	0.99	1.82	0.75	1.06	0.64	0.52	1.05	1.05
	B110.3	P	0.49	1.03	1.91	0.79	1.11	0.67	0.55	1.10	1.10
	B5UR1	P	1.18	1.07	1.21	1.34	0.87	1.11	1.03	1.07	1.00
** P—Plain section; H—Hollow section.											

**Ref.**	**Beam**	******	$\frac{T_{R,exp}}{T_{R,th}^{SiNiP18}}$	$\frac{T_{R,exp}}{T_{R,th}^{ACI89}}$	$\frac{T_{R,exp}}{T_{R,th}^{ACI19}}$	$\frac{T_{R,exp}}{T_{R,th}^{MC90}}$	$\frac{T_{R,exp}}{T_{R,th}^{MC10}}$	$\frac{T_{R,exp}}{T_{R,th}^{EC2}}$	$\frac{T_{R,exp}}{T_{R,th}^{CSA14}}$	$\frac{T_{R,exp}}{T_{R,th}^{Rahal}}$	$\frac{T_{R,exp}}{T_{R,th}^{Prop}}$
Koutchoukali, Belarbi (2001)	B7UR1	P	1.12	0.92	1.03	1.26	0.74	1.05	0.97	0.94	0.85
	B9UR1	P	1.29	1.05	1.19	1.45	0.85	1.21	1.12	1.05	0.94
	B12UR1	P	1.15	0.91	1.05	1.29	0.76	1.08	1.00	0.93	0.84
	B12UR2	P	1.08	0.86	0.99	1.21	0.71	1.01	0.94	0.89	0.80
	B12UR3	P	1.18	1.01	1.04	1.28	0.75	1.06	0.99	0.98	0.89
	B12UR4	P	1.24	1.02	1.07	1.22	0.71	1.01	0.94	0.96	0.88
	B12UR5	P	0.93	0.86	1.08	1.08	0.64	0.90	0.82	0.87	0.80
	B14UR1	P	1.26	0.98	1.16	1.42	0.83	1.18	1.10	1.00	0.88
Fang, Shiau (2004)	H-06-06	P	1.59	1.08	1.21	1.59	0.81	1.31	1.15	1.05	0.97
	H-06-12	P	1.74	1.36	1.19	1.57	0.85	1.29	1.13	1.12	1.04
	H-07-10	P	1.46	1.48	1.27	1.67	0.98	1.38	1.21	1.23	1.15
	H-07-16	P	1.18	1.69	1.27	1.48	1.32	1.22	1.07	1.17	1.12
	H-12-12	P	1.93	1.16	1.28	1.50	0.98	1.24	1.08	1.18	1.12
	H-12-16	P	1.40	1.47	1.61	1.43	1.34	1.18	1.03	1.23	1.19
	H-14-10	P	1.38	1.09	1.19	1.35	0.90	1.11	0.97	1.08	1.03
	H-20-20	P	1.43	1.33	1.97	1.19	1.51	0.99	0.86	1.43	1.43
	N-06-06	P	1.38	1.09	1.05	1.38	0.99	1.14	0.99	1.03	0.99
	N-06-12	P	1.44	1.30	1.16	1.30	1.36	1.07	0.94	1.05	1.02
	N-07-10	P	1.29	1.48	1.41	1.47	1.63	1.22	1.06	1.21	1.19
	N-07-16	P	0.96	1.56	1.48	1.20	2.02	0.99	0.87	1.07	1.06
	N-12-12	P	1.45	0.96	1.43	1.13	1.42	0.93	0.81	1.01	1.01
	N-12-16	P	0.98	1.14	1.69	1.01	1.82	0.83	0.75	1.05	1.05
	N-14-10	P	1.28	1.10	1.57	1.25	1.56	1.03	0.90	1.12	1.11
	N-20-20	P	0.94	1.30	1.93	1.02	1.92	0.81	0.86	1.20	1.20
Chiu et al. (2007)	HAS-51-50	P	2.10	1.15	1.55	2.24	1.02	1.68	1.47	1.24	1.11
	NAS-61-35	P	1.55	1.33	1.49	2.15	1.00	1.61	1.41	1.25	1.14
	HAS-90-50	P	1.64	1.40	1.43	2.06	0.96	1.55	1.36	1.24	1.13
	NBS-43-44	P	1.77	1.11	1.32	1.74	0.88	1.44	1.26	1.13	1.05
	HBS-74-17	P	0.95	1.19	1.18	1.55	0.79	1.28	1.12	0.94	0.85
	HBS-82-13	P	0.82	1.19	1.20	1.57	0.80	1.30	1.14	0.92	0.83
	NBS-82-13	P	0.77	1.39	1.12	1.48	1.11	1.22	1.07	0.96	0.90
	HBS-60-61	P	1.79	1.23	1.47	1.93	0.99	1.59	1.40	1.24	1.14
	HCS-52-50	P	1.83	1.16	1.64	1.97	1.12	1.79	1.55	1.14	1.02
	HCS-91-50	P	1.61	1.50	1.60	1.92	1.11	1.75	1.52	1.21	1.10
Lee and Kim (2010)	T1-1	P	1.41	1.06	1.25	1.71	0.85	1.34	1.18	1.11	1.02
	T1-2	P	1.64	1.14	1.15	1.57	0.91	1.24	1.09	1.16	1.09
	T1-3	P	1.75	1.13	1.30	1.40	1.12	1.10	0.97	1.13	1.09
	T1-4	P	1.15	0.97	1.49	1.10	1.22	0.87	0.76	0.99	0.98
	T2-2	P	0.91	1.24	1.04	1.42	0.90	1.11	0.98	1.03	0.96
	T2-3	P	1.12	1.28	1.19	1.25	1.17	0.98	0.87	1.02	0.98
	T2-4	P	0.90	1.30	1.34	1.20	1.30	0.94	0.83	1.02	0.99
	SW12-1	P	1.11	1.71	1.86	1.52	1.25	1.47	1.28	0.85	0.81
** P—Plain section; H—Hollow section.											

**Ref.**	**Beam**	******	$\frac{T_{R,exp}}{T_{R,th}^{SiNiP18}}$	$\frac{T_{R,exp}}{T_{R,th}^{ACI89}}$	$\frac{T_{R,exp}}{T_{R,th}^{ACI19}}$	$\frac{T_{R,exp}}{T_{R,th}^{MC90}}$	$\frac{T_{R,exp}}{T_{R,th}^{MC10}}$	$\frac{T_{R,exp}}{T_{R,th}^{EC2}}$	$\frac{T_{R,exp}}{T_{R,th}^{CSA14}}$	$\frac{T_{R,exp}}{T_{R,th}^{Rahal}}$	$\frac{T_{R,exp}}{T_{R,th}^{Prop}}$
Peng, Wong (2011)	SW10-1	P	0.93	1.49	1.92	1.28	1.65	1.23	1.07	0.79	0.77
	SW10-2	P	1.17	1.37	2.19	1.30	1.15	1.25	1.05	0.89	0.89
	SW10-3	P	1.19	1.83	2.93	1.12	1.86	1.08	1.44	1.10	1.10
	SW10-4	P	1.06	1.85	3.23	0.92	1.82	0.88	1.48	1.14	1.14
	SW8-1	P	1.32	1.53	1.91	1.31	1.64	1.25	1.15	0.90	0.87
	SW8-2	P	1.74	1.85	3.08	1.20	1.90	1.14	1.51	1.15	1.15
Joh et al. (2019)	RA-SD4-3.2-0.3-3.28	P	0.89	1.47	1.15	1.17	1.42	0.95	0.79	1.00	0.96
	RA-SD5-3.2-0.3-3.21	P	0.85	1.48	1.16	1.13	1.49	0.91	0.76	0.98	0.94
	RA-SD6-3.2-0.2-3.21	P	0.71	1.51	1.18	1.02	1.66	0.83	0.68	0.96	0.96
	RA-SD4-3.2-0.5-2.13	P	1.20	1.26	0.98	1.38	0.95	1.12	0.93	1.06	0.99
	RA-SD5-3.2-0.7-1.63	P	1.39	1.23	1.06	1.49	0.90	1.21	1.00	1.11	1.03
	RA-SD6-3.2-0.6-1.63	P	1.14	1.15	0.91	1.29	0.89	1.05	0.87	0.98	0.92
	RA-SD4-3.2-1.1-1.33	P	2.10	1.23	1.40	1.98	0.94	1.61	1.33	1.35	1.23
	RA-SD5-3.2-1.0-1.26	P	1.79	1.12	1.21	1.71	0.81	1.39	1.15	1.19	1.09
	RA-SD6-3.2-0.8-1.26	P	1.29	1.15	1.12	1.58	0.81	1.28	1.06	1.13	1.04
Ju et al. (2019)	MR30-0.77	P	1.40	1.08	1.12	1.47	0.99	1.21	1.06	1.00	0.94
	MT30-1.32	P	0.71	1.12	0.77	1.00	1.30	0.83	0.72	0.77	0.75
	MT40-1.32	P	0.73	1.07	0.78	1.02	0.90	0.85	0.74	0.75	0.72
	MT40-1.89	P	1.05	0.92	1.13	1.05	1.09	0.86	0.75	0.89	0.88
Ibrahim et al. (2020)	NSC-S1-C30	P	0.81	0.85	1.16	0.95	0.94	0.79	0.71	0.85	0.81
	NSC-S1-C45	P	0.52	0.67	1.23	0.74	0.59	0.62	0.57	0.55	0.52
	HSC-C30	P	0.82	0.82	0.97	0.96	0.71	0.80	0.71	0.81	0.75
	HSC-C45	P	0.57	0.70	1.09	0.82	0.48	0.68	0.63	0.56	0.52
Kim et al. (2020)	S08-3-65	P	1.73	1.14	1.28	1.48	0.86	1.24	1.22	1.07	1.04
	S08-4-90	P	1.95	1.12	1.21	1.38	0.83	1.15	1.14	1.03	0.99
	S08-5-122.5	P	1.10	0.86	0.93	1.08	0.62	0.90	0.89	0.78	0.75
	S10-3-52.5	P	1.62	1.01	1.23	1.22	0.84	1.01	1.00	0.94	0.92
	S10-4-72.5	P	1.09	0.85	1.06	0.98	0.73	0.82	0.81	0.78	0.76
	S10-5-100	P	1.20	0.91	1.05	1.06	0.73	0.89	0.87	0.82	0.80
	S06-3-90	P	2.34	1.14	1.43	1.66	0.96	1.38	1.36	1.09	1.03
	S10-5-90	P	1.10	0.83	1.03	0.94	0.72	0.78	0.77	0.75	0.73
	S08-3-72.5	P	1.59	1.05	1.23	1.42	0.82	1.18	1.17	1.00	0.95
	S12-5-72.5	P	0.97	0.80	1.17	0.85	0.81	0.71	0.70	0.72	0.72
Hsu (1968)	D3	H	0.59	1.44	1.38	1.49	1.65	1.24	1.09	1.22	1.00
	D4	H	0.64	1.06	1.63	1.07	1.62	0.89	0.78	1.01	1.00
[17,38]	T0	H	1.59	2.03	1.65	1.86	1.33	1.39	1.27	1.26	1.15
	T1	H	1.98	1.32	1.27	1.67	1.06	1.25	1.05	1.12	1.09
	T2	H	1.93	1.64	1.44	1.88	1.12	1.41	1.28	1.23	1.11
	T5	H	1.79	0.81	1.16	1.44	0.72	1.26	1.10	0.95	0.84
[19]	VH1	H	1.70	1.04	1.08	1.72	1.50	1.29	1.03	1.11	1.05
	VH2	H	1.35	0.75	1.05	1.14	2.22	0.83	0.67	0.69	1.07
	A1	H	1.39	1.23	1.48	2.22	1.00	1.66	1.41	1.12	1.02
** P—Plain section; H—Hollow section.											

**Ref.**	**Beam**	******	$\frac{T_{R,exp}}{T_{R,th}^{SiNiP18}}$	$\frac{T_{R,exp}}{T_{R,th}^{ACI89}}$	$\frac{T_{R,exp}}{T_{R,th}^{ACI19}}$	$\frac{T_{R,exp}}{T_{R,th}^{MC90}}$	$\frac{T_{R,exp}}{T_{R,th}^{MC10}}$	$\frac{T_{R,exp}}{T_{R,th}^{EC2}}$	$\frac{T_{R,exp}}{T_{R,th}^{CSA14}}$	$\frac{T_{R,exp}}{T_{R,th}^{Rahal}}$	$\frac{T_{R,exp}}{T_{R,th}^{Prop}}$
Bernardo, Lopes (2009)	A2	H	1.75	1.31	1.20	1.76	0.81	1.32	1.14	1.12	1.07
	A3	H	1.62	1.25	1.25	1.56	0.94	1.17	1.01	1.08	1.07
	A4	H	1.37	1.26	1.55	1.38	1.04	1.04	0.92	1.03	1.02
	A5	H	1.27	1.23	1.73	1.25	1.22	0.94	0.83	1.00	1.01
	B2	H	1.80	1.26	1.22	1.79	0.83	1.34	1.16	1.09	1.02
	B3	H	1.32	1.11	1.14	1.33	0.75	1.00	0.86	0.94	0.92
	B4	H	1.29	1.17	1.38	1.26	0.91	0.94	0.83	0.96	0.95
	B5	H	1.10	1.05	1.47	1.05	0.96	0.79	0.71	0.92	0.92
	C1	H	1.39	1.03	1.48	2.23	1.00	1.68	1.40	1.02	0.90
	C2	H	1.83	1.23	1.26	1.84	0.83	1.38	1.20	1.05	0.97
	C3	H	1.34	1.12	1.08	1.36	0.69	1.02	0.86	0.93	0.89
	C4	H	1.39	1.20	1.36	1.37	0.89	1.03	0.86	1.00	0.98
	C5	H	1.15	1.07	1.38	1.17	0.88	0.88	0.74	0.90	0.89
	C6	H	1.07	1.12	1.69	1.00	1.06	0.75	0.65	1.01	1.01
Chiu et al. (2007)	HAH-81-35	H	1.37	1.78	1.46	2.11	0.97	1.58	1.39	1.22	1.11
	NCH-62-33	H	1.61	1.41	1.60	1.93	1.11	1.75	1.52	1.16	1.06
	HCH-91-42	H	1.55	1.51	1.59	1.91	1.10	1.74	1.51	1.17	1.06
Jeng (2014)	A095c	H	1.13	1.47	1.94	2.63	1.53	2.17	1.84	1.56	1.24
	A120a	H	1.08	2.22	2.12	3.29	1.92	2.72	2.01	1.73	1.17
	B065b	H	1.06	2.17	1.48	1.59	1.83	1.31	1.11	1.17	0.97
	B080a	H	1.06	1.24	1.20	1.52	0.97	1.25	1.06	1.12	1.10
	B110a	H	1.12	1.86	2.02	2.84	1.65	2.34	1.92	1.59	1.14
	C100a	H	1.06	1.20	1.27	1.72	0.94	1.43	1.21	1.17	1.11
	D075a	H	1.06	1.35	1.25	1.69	0.81	1.40	1.18	1.11	1.05
	D090a	H	1.05	1.15	1.18	1.60	0.81	1.32	1.12	1.06	0.99
Kim et al. (2020)	H08-3-65	H	1.60	1.14	1.23	1.40	0.87	1.17	1.15	1.04	1.01
	H08-4-90	H	1.75	1.22	1.35	1.56	0.89	1.30	1.28	1.12	1.08
	H08-5-100	H	1.53	1.05	1.12	1.29	0.80	1.07	1.06	0.95	0.92
	H10-3-52.5	H	1.55	1.10	1.37	1.27	0.97	1.06	1.05	1.00	0.99
	H10-4-72.5	H	1.35	1.04	1.22	1.22	0.87	1.02	1.01	0.94	0.92
	H10-5-80	H	1.33	0.96	1.20	1.12	0.85	0.93	0.92	0.88	0.86
	H06-3-90	H	2.15	1.12	1.33	1.54	0.83	1.28	1.27	1.04	0.98
	H10-5-135	H	1.35	1.02	1.20	1.39	0.75	1.16	1.14	0.95	0.90
	H08-3-72.5	H	1.51	1.09	1.19	1.38	0.78	1.15	1.13	0.99	0.95
	H12-5-72.5	H	1.38	0.93	1.24	1.19	0.84	0.99	0.98	0.93	0.91
** P—Plain section; H—Hollow section.											
